# Utilizing MiSeq Sequencing to Detect Circulating microRNAs in Plasma for Improved Lung Cancer Diagnosis

**DOI:** 10.3390/ijms241210277

**Published:** 2023-06-17

**Authors:** Xinyan Geng, Jen-Hui Tsou, Sanford A. Stass, Feng Jiang

**Affiliations:** Department of Pathology, University of Maryland School of Medicine, 10 South Pine Street, MSTF 7th Floor, Baltimore, MD 21201-1192, USA

**Keywords:** NGS, circulating cell-free, miRNAs, lung cancer, diagnosis

## Abstract

Non-small cell lung cancer (NSCLC) is a major contributor to cancer-related deaths, but early detection can reduce mortality. NSCLC comprises mainly adenocarcinoma (AC) and squamous cell carcinoma (SCC). Circulating microRNAs (miRNAs) in plasma have emerged as promising biomarkers for NSCLC. However, existing techniques for analyzing miRNAs have limitations, such as restricted target detection and time-consuming procedures. The MiSeqDx System has been shown to overcome these limitations, making it a promising tool for routine clinical settings. We investigated whether the MiSeqDx could profile cell-free circulating miRNAs in plasma and diagnose NSCLC. We sequenced RNA from the plasma of patients with AC and SCC and from cancer-free smokers using the MiSeqDx to profile and compare miRNA expressions. The MiSeqDx exhibits high speed and accuracy when globally analyzing plasma miRNAs. The entire workflow, encompassing RNA to data analysis, was completed in under three days. We also identified panels of plasma miRNA biomarkers that can diagnose NSCLC with 67% sensitivity and 68% specificity, and detect SCC with 90% sensitivity and 94% specificity, respectively. This study is the first to demonstrate that rapid profiling of plasma miRNAs using the MiSeqDx has the potential to offer a straightforward and effective method for the early detection and classification of NSCLC.

## 1. Introduction

Lung cancer is the second most commonly diagnosed cancer and the leading cause of cancer-related deaths worldwide [1]. Non-small cell lung cancer (NSCLC) constitutes 85% of all types of lung cancer cases [2]. Adenocarcinoma (AC) and squamous cell cancer (SCC) are two major histological types of NSCLC. Patients with early stage lung cancer have a better prognosis compared to those with advanced-stage lung cancer, who lose the opportunity to receive effective treatments including radical surgery. For patients with regional disease, the 5-year survival rate is 33%, while in patients with distant metastasis, it is only 6% [2]. Hence, early detection is crucial in improving the prognosis of lung cancer. Currently, low-dose CT (LDCT) screening is the gold standard in diagnosing early stage lung cancer. However, LDCT is limited as it cannot distinguish between benign and malignant pulmonary nodules. For instance, only 4% of patients with pulmonary nodules detected via LDCT are eventually diagnosed with early stage lung cancer, while the remaining 96% have benign nodules. Although bronchoscopy can accurately diagnose the abnormal area, it is an invasive and harmful procedure. Therefore, there is an urgent need for new, noninvasive methods to diagnose lung cancer accurately and efficiently at the curable stages.

MicroRNAs (miRNAs) are a class of small non-coding RNAs with a length of ~22 nucleotides. miRNAs are mainly involved in suppressing gene expression at the post-transcriptional level, while under certain circumstances, miRNA can upregulate gene expression and regulate gene transcription [3,4,5,6,7]. Furthermore, miRNAs play important roles in regulating physiological and pathological processes, such as developmental timing, cell proliferation, apoptosis, aging, cancer, etc. [8,9,10,11]. In addition, miRNAs can be secreted into extracellular fluids and transported to remote targeting cells via vesicles or by binding to proteins [12,13]. Previous studies including our own have shown that circulating microRNAs in plasma can be promising biomarkers for lung cancer diagnosis [14,15,16,17,18,19,20,21].

Techniques such as polymerase chain reaction (PCR) and whole genome next-generation sequencing (WGS) are commonly used to detect miRNAs. PCR is a cost-effective and rapid method for measuring miRNA expression levels; however, it only covers a limited number of targets at once. WGS allows for the evaluation of whole genomes, and thus generates large amounts of data for high-throughput analysis [22]. However, it is expensive, technically complex, and requires extensive data analysis. It may also have limited sensitivity and generate false positives, restraining its applicability in routine clinical settings [22]. New techniques that can rapidly and sensitively quantify a large number of miRNAs while remaining cost-effective are urgently needed.

The Food and Drug Administration granted marketing authorization for the first high-throughput next-generation sequencing (NGS) instrument, Illumina’s MiSeqDx [23,24]. The MiSeq utilizes Illumina’s sequencing-by-synthesis technology, which involves the cyclic addition and detection of fluorescently labeled nucleotides. It is a benchtop sequencer that enables for targeted resequencing, metagenomics, small genome sequencing, amplicon sequencing, and other applications. The MiSeq instrument offers a relatively compact and cost-effective solution for laboratories that require medium-throughput sequencing. Furthermore, it can process a range of sample types, from DNA extracted from cells or tissues to amplicons generated via PCR amplification. The system provides flexibility in terms of read lengths and can generate data outputs suitable for various downstream analysis applications. In addition, designed specifically for the clinical laboratory environment, the MiSeqDx instrument offers a small footprint, an easy-to-use workflow, and data output tailored to the diverse needs of clinical labs. Compared with conventional PCR, the MiSeqDx instrument has several advantages, including the ability to analyze multiple targets at once, lower cost per sample, higher throughput, and more comprehensive data output [22]. Compared to WGS, the MiSeqDx instrument offers cost-effectiveness and a faster turnaround time, making it suitable for clinical applications. Moreover, the MiSeqDx System has a high sensitivity for detecting low-abundance molecules and a high specificity for reducing false positives, which are both desirable qualities in an analytical technique [23]. Additionally, the MiSeqDx instrument has been used as in vitro diagnostic (IVD) testing assays in the clinical laboratory by detecting the disease-related DNA mutations [24]. Yet, the use of the MiSeqDx instrument in the analysis of circulating miRNAs for cancer diagnosis remains to be investigated.

Our objective was to examine if the MiSeqDx instrument would be capable of profiling cell-free circulating miRNAs in plasma and being used as a potential tool for the diagnosis of lung cancer.

## 2. Results

### 2.1. The Assessment of Hemolysis in Plasma

Hemolysis of blood samples can cause the release of red blood cell-derived miRNAs into plasma, leading to non-specific and low-reproducibility outcomes in the quantification of cancer-related miRNAs [25]. To determine if the plasma samples have hemolysis of blood cells, we tested hemolysis-associated miRNA markers (miR-23a-3p and miR-451a) in the plasma samples by using qRT-PCR. These microRNAs are enriched in red blood cells and are released into the plasma when red blood cells are lysed [25]. The difference in qPCR quantitative cycles (Cq) values between the two miRNAs is known to increase in the presence of hemolysis, which can be used as an indicator of hemolysis in plasma samples. The specimens with ΔCq (miR-23a-3p–miR-451a) higher than 8.0 are considered to have hemolysis [25]. In this study, all plasma specimens exhibited ≤ΔCq 8.0 values, indicating the absence of hemolysis. Subsequently, these specimens underwent processing for MiSeq NGS.

### 2.2. Small RNA Sequencing, Quality Control, and Annotation

To evaluate the sequencing quality, we conducted an analysis of the raw reads obtained from the sequenced libraries of the plasma samples. The total reads of the samples ranged from 4.2 to 8.3 million (Supplementary Table S1). Furthermore, the base calling accuracy was high, with the average Phred quality score (Q score) of the Unique Molecular Index (UMI) reads being at least 38. In addition, the majority of the plasma specimens had a Q score above 40. Therefore, despite the variability in the number of UMI reads across the samples, the high-throughput MiSeq sequencing of multiple samples provided sufficient and accurate data to allow for detailed analysis [26]. Other key quality control metrics, including the number of discarded reads, UMI groups, merged UMI groups, average Q score of UMI reads, and average reads per UMI (Supplementary Table S2) also indicated that the resulting UMI reads were of high quality and suitable for subsequent analysis.

We then mapped the raw reads to a human genome. A total of 1917 annotated miRNAs in humans were listed by miRbase. The annotated miRNAs identified in the samples ranged from 422 to 953, with a median of 706. The number of reads annotated with miRBase ranged from 210,569 (27.64%) to 2,881,601 (79.49%) (Supplementary Tables S3–S5). Therefore, the MiSeq sequencing approach was able to detect a large number of miRNAs and provided a useful resource for interpretation of the sequencing data.

Principal component analysis (PCA) was then utilized to evaluate both the inter- and intragroup variability of miRNA expression in the plasma of the cancer patients and cancer-free controls. The resulting plots exhibited different miRNAs profiles among the patient groups and control group (Supplementary Figure S1). Furthermore, some of the miRNAs were also found to be associated with patients’ demographic characteristics, such as age, gender, race, and smoking status, as determined via an ANOVA test (Supplementary Table S6). In addition, the streamlined workflow of the MiSeqDx could process RNA samples with high efficiency, generate data, and perform final data analysis all within a timeframe of fewer than three days.

### 2.3. Differently Expressed Plasma miRNAs between NSCLC Patients and Cancer-Free Smokers

**Figure 1:**
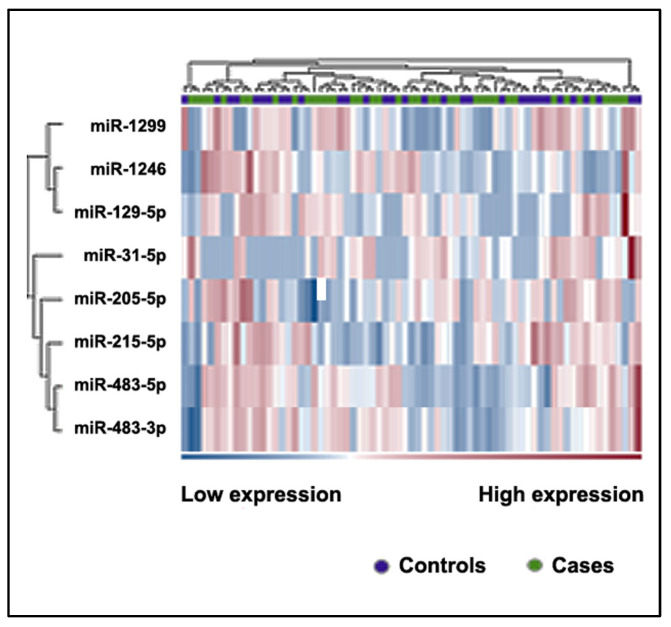
Heatmap of differentially expressed miRNAs in the plasma of patients with NSCLC and controls. The heatmap shows only those miRNAs with an FC greater than 2 and a false discovery rate (FDR) of *p*-value ≤ 0.1. The color scale bar represents the relative expression of the miRNAs, with red indicating upregulation and blue indicating downregulation.

Eight miRNAs had a fold change (FC) of at least 2 with a *p*-value ≤ 0.0001 between NSCLC patients and controls (Table 1 and Figure 1, Supplementary Figure S2).

**Figure 2:**
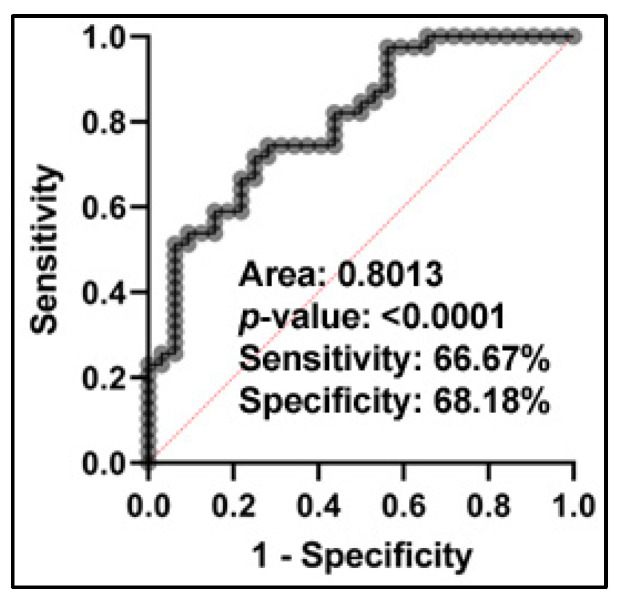
Receiver Operating Characteristic (ROC) curve analysis of the combined four plasma miRNAs in distinguishing patients with NSCLC from non-cancer controls. The ROC curve demonstrates the performance of the panel of four plasma miRNAs in distinguishing between NSCLC patients and controls, with the area under the curve (AUC) value indicating the overall diagnostic accuracy. The analysis shows that the panel of four plasma miRNAs have 0.80 AUC, with a sensitivity of 66.7% and specificity of 68.2%.

From the eight miRNAs, we identified four miRNAs (miRs-215-5p, 1299, 205-5p, and 1246) as the best combination in classifying NSCLC patients from non-cancer controls using LASSO regression (Supplementary Figure S3). The predicting algorithm of NSCLC from the non-cancer controls was as follows: Log (probability of NSCLC/probability of non-cancer control) = 0.4718 + 0.00129 * miR-1246 − 0.02465 * miR-1299 + 0.00896 * miR-205-5p − 0.03244 * miR-215-5p. Combined analysis of the four miRNAs yielded 0.8013 AUC *p* = 0.0001 (Figure 2). As a result, the four miRNAs used in combination created 66.67% sensitivity and 68.18% specificity in diagnosing NSCLC. miRNA-205-5p is associated with the stage of lung tumors, with higher plasma expression levels in advanced-stage lung cancer (*p* = 0.003, ANOVA test) (Supplementary Table S6). However, combining the four miRNAs as a panel of biomarkers did not show a significant association with tumor stage (*p* > 0.05). The results suggest that the plasma miRNA biomarker panel may have potential for the early detection of NSCLC. Pearson correlation analysis of the four miRNAs indicated no significant collinearity among these miRNAs (Supplementary Table S7 and Figure S4), further implying that the integration of the different miRNAs biomarkers provides complementary diagnosis of NSCLC.

### 2.4. Differently Expressed Plasma miRNAs between SCC Patients and Cancer-Free Smokers

**Figure 3:**
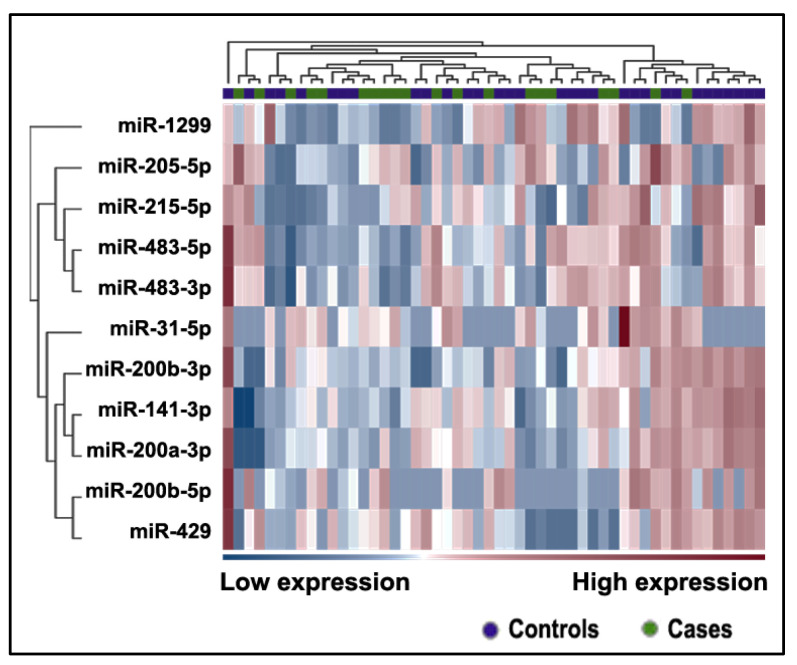
Heatmap of differentially expressed miRNAs in the plasma of patients with SCC and controls. The heatmap shows only those miRNAs with an FC greater than 2 and an FDR *p*-value ≤ 0.1. The color scale bar represents the relative expression of the miRNAs, with red indicating upregulation and blue indicating downregulation.

Eleven miRNAs displayed abnormal expression levels in the plasma of SCC patients in comparison to cancer-free smokers (Table 2 and Figure 3) (Supplementary Figure S5) (all *p* < 0.0001).

**Figure 4:**
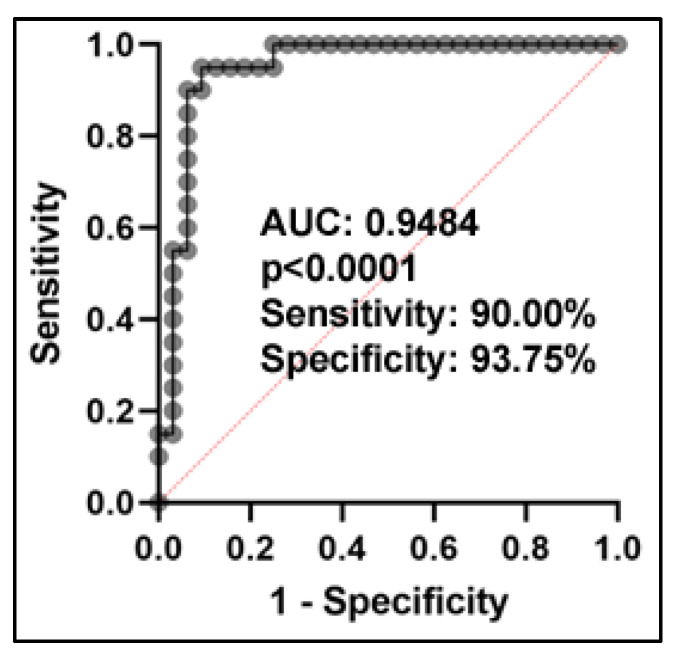
ROC curve analysis of combined five plasma miRNAs in distinguishing patients with NSCLC from non-cancer controls. The ROC curve demonstrates the performance of the panel of five miRNAs in distinguishing between SCC patients and controls, with the AUC value indicating the overall diagnostic accuracy. The analysis shows that the panel of five plasma miRNAs have 0.95 AUC, with a sensitivity of 90.0% and specificity of 93.8%.

From the eleven miRNAs, we identified a set of five miRNAs (miRs-205-5p, 1299, 215-5p, 141-3p and 200b-5p) as the best combination in classifying SCC patients from non-cancer controls using LASSO regression (Supplementary Figure S6). The prediction algorithm with the five miRNAs was formulated as follows: Log (probability of SCC/probability of non-cancer control) = 0.8825 + 0.03495 * miR-205-5p − 0.1203 * miR-1299 − 0.06752 * miR-215-5p − 0.02708 * miR-141-3p − 0.6753 * miR-200b-5p. Combined analysis of the five miRNAs yielded 0.948 AUC with 90.00% sensitivity and 93.75% specificity in diagnosing SCC (*p* = 0.0001) (Figure 4). Furthermore, integrating the five miRNAs as a panel of biomarkers did not demonstrate a significant association with tumor stage (*p* > 0.05), suggesting the potential for the early detection of SCC. No significant collinearity was observed among the miRNAs (Supplementary Figure S7 and Table S8), indicating that the integration of different miRNA biomarkers provides complementary diagnostic information.

### 2.5. Differently Expressed Plasma miRNAs between AC Patients and Cancer-Free Smokers

**Figure 5:**
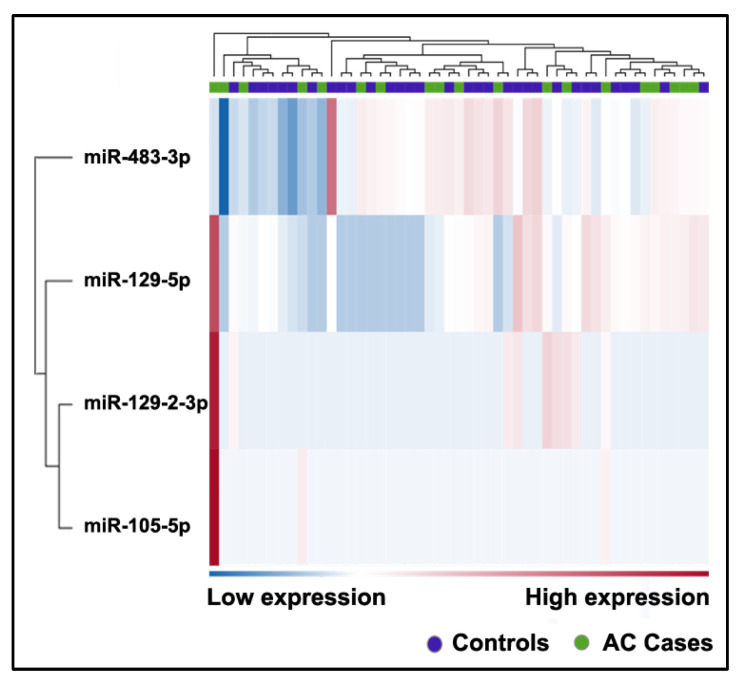
Heatmap of differentially expressed miRNAs in the plasma of patients with AC and controls. The heatmap shows only those miRNAs with an FC greater than 2 and an FDR *p*-value ≤ 0.1. The color scale bar represents the relative expression of the miRNAs, with red indicating upregulation and blue indicating downregulation.

Four miRNAs with an FC greater than 2 (a *p*-value ≤ 0.0001) were identified as differentially expressed between the AC group and the control group (Table 3, Figure 5, Supplementary Figure S8).

Two miRNAs, miR-105-5p and miR-129-2-3p, were excluded from further analysis as they were expressed in less than half of the plasma samples from either cancer patients or non-cancer controls. We conducted a comparison of miR-129-5p and miR-483-3p expression levels between cancer patients and non-cancer controls. These two miRNAs exhibited an AUC ≥ 0.5 for discriminating AC patients from non-cancer controls (Supplementary Figure S9). However, the statistical power of miR-129-5p and miR-483-3p in discriminating between AC patients and non-cancer controls did not reach significance with *p* ≤ 0.05. Therefore, none of the plasma miRNAs analyzed in this study demonstrated potential for diagnosing patients with AC.

### 2.6. Differently Expressed Plasma miRNAs between AC Patients and SCC Patients

**Figure 6:**
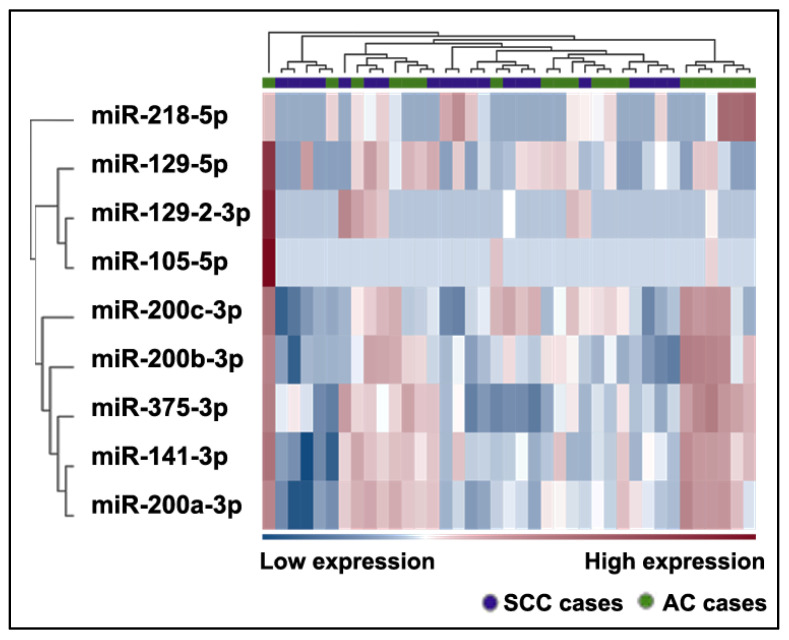
Heatmap of differentially expressed miRNAs in the plasma of patients with AC and SCC. The heatmap shows only those miRNAs with an FC greater than 2 and an FDR *p*-value ≤ 0.1. The color scale bar represents the relative expression of the miRNAs, with red indicating upregulation and blue indicating downregulation.

Nine miRNAs were identified as downregulated in SCC compared to AC, with a fold change greater than 2 (*p* ≤ 0.0001) (Table 4, Figure 6, Supplementary Figure S10).

**Figure 7:**
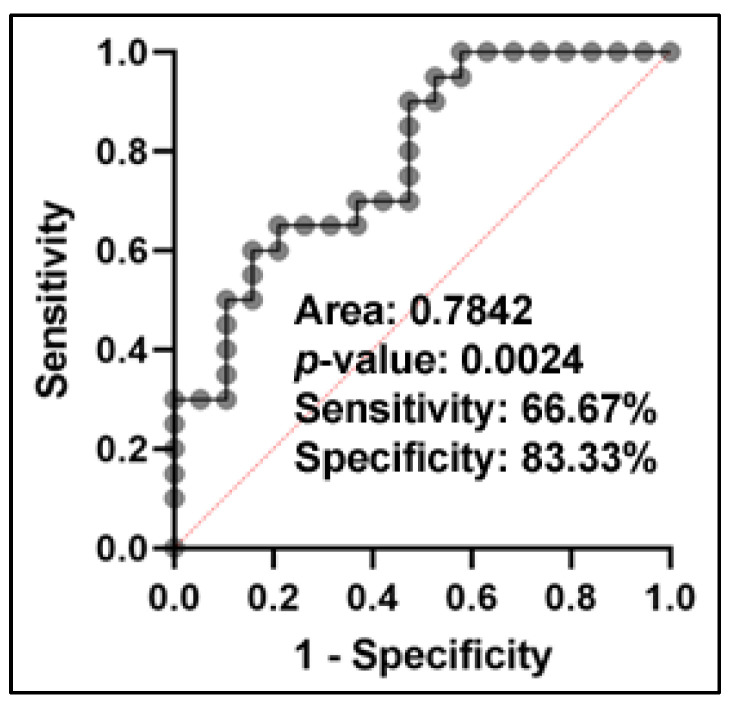
An ROC curve analysis was performed to evaluate the performance of a combination of two plasma miRNAs in distinguishing patients with SCC from AC. The ROC curve demonstrates the overall diagnostic accuracy of the panel of two miRNAs, with the AUC value indicating the degree of separation between SCC and AC patients. The analysis showed that the panel of two miRNAs had an AUC of 0.78, with a sensitivity of 66.7% and specificity of 83.3% for distinguishing between SCC and AC patients.

Using LASSO regression, we identified miR-200a-3p and miR-218-5p as the best combination of miRNAs for classifying AC from SCC (Supplementary Figure S11). The logarithm of the probability is 1.614 − 0.05023 * miR-200a-3p − 0.09593 * miR-218-5p. The panel of two miRNAs showed a predictive capacity for distinguishing SCC from AC patients, resulting in an AUC of 0.78 (*p* = 0.001) with 66.7% sensitivity and 83.33% specificity (Figure 7).

Furthermore, no significant correlation was found between miRs-200a-3p and 218-5p, indicating the synergetic effect of their combination in differentiating SSC from AC (Supplementary Figure S12 and Table S9).

## 3. Discussion

Although PCR is an easy technique for assessing plasma miRNAs, it only covers a limited number of targets at once. On the other hand, WGS allows for the evaluation of whole genomes, producing vast amounts of data for high-throughput analysis. However, it is technically complex and involves extensive data analysis. In this study, we demonstrated that the MiSeqDx platform exhibited exceptional performance, allowing for the generation of data within a remarkably short timeframe of less than three days. The MiSeqDx instrument can significantly reduce the turnaround time compared to with WGS demanding at least 10 days, making it a highly valuable tool for research and clinical settings that require rapid results. Furthermore, the high-throughput MiSeqDx sequencing of multiple samples provided sufficient and accurate data to allow for detailed analysis of a large number of miRNAs. Therefore, the MiSeqDx platform has the advantage of rapidly quantifying multiple miRNAs simultaneously, overcoming the limitations of PCR and WGS platforms. Additionally, as the MiSeqDx platform has already been utilized as an IVD testing assay in the clinical laboratory to detect disease-related DNA mutations [24], it may be developed as an IVD assay to identify disease-related miRNAs in the clinical laboratory.

While the primary goal of this study was to investigate the benefits of MiSeqDx for future clinical applications, we have also made an exciting discovery in plasma miRNA biomarkers. Using LASSO regression analysis of the MiSeqDx data, we identified three panels of plasma miRNAs biomarkers for diagnosis and classification of NSCLC: a panel of miRs-215-5p, 1299, 205-5p, and 1246 that can detect NSCLC with 67% sensitivity and 68% specificity; a panel of miRs-205-5p, 1299, 215-5p, 141-3p, and 200b-5p that can detect SCC with 90% sensitivity and 94% specificity; and a panel of miRs-200a-3p and 218-5p that can distinguish SCC from AC with 67% sensitivity and 83% specificity. Furthermore, Pearson correlation analysis revealed no significant correlation between the plasma miRNAs in each panel of the biomarkers, implying that their diagnostic values were complementary to each other. In addition, the plasma miRNAs used in each panel showed independent diagnostic values across different tumor stages, indicating their potential effectiveness in detecting and grouping the major types of NSCLC at early stages. Therefore, rapid profiling of plasma miRNAs using the MiSeqDx method may provide an efficient method for the early diagnosis and classification of lung cancer.

MiR-205-5p is increased in lung cancer, particularly in squamous cell carcinoma (SCC) of the lung, and promotes tumor growth and metastasis by regulating cell signaling pathways [27,28]. Elevated levels of miR-205-5p are associated with poor prognosis and reduced overall survival in lung cancer patients. Previous studies, including our own, have suggested that miR-205-5p could serve as a potential biomarker for the early detection of lung cancer and classification of different types of NSCLC [29,30,31]. miR-215-5p has been suggested to act as a tumor suppressor by regulating cell proliferation, invasion, and migration [32]. Low expression of miR-215-5p in tumor tissues has been associated with poor prognosis and reduced overall survival rates, indicating its potential as a diagnostic biomarker for breast cancer [27]. miRNA-1246 can suppress the proliferation and migration of renal cell carcinoma through targeting CXCR4 [28] and has potential as a circulating biomarker for multiple myeloma [33]. miR-1299 has both tumor-suppressive and oncogenic roles in different types of cancers [34]. It has been shown to inhibit cancer cell proliferation, invasion, and migration in some cancers, such as colorectal cancer, but promote these processes in other cancers, such as breast cancer [34]. Low expression of miR-1299 has been associated with poor prognosis and reduced overall survival rates in colorectal cancer, while high expression has been linked to poor prognosis in breast cancer [34]. miR-141-3p could inhibit cancer cell proliferation, invasion, and migration in prostate cancer, but could promote these processes in breast cancer as well as act as a potential biomarker for gastric cancer [35]. miR-200b-5p and miR-200a-3p act as tumor suppressors or oncogenes, depending on the cancer type [36]. Targeting miR-200b-5p and miR-200a-3p has been shown to inhibit cancer cell growth and metastasis in preclinical studies [36]. miR-218-5p functions as a tumor suppressor by inhibiting cancer cell growth and metastasis [37]. It has been shown to target genes involved in epithelial–mesenchymal transition and angiogenesis in lung cancer and hepatocellular carcinoma [38]. Furthermore, miR-218-5p affects lung adenocarcinoma progression through targeting endoplasmic reticulum oxidoreductase 1 alpha [39]. Our findings of the differential levels of the miRNAs in the plasma of NSCLC patients not only confirm their significant roles in lung carcinogenesis, but also provide compelling evidence for their potential as circulating biomarkers for the diagnosis and classification of lung cancer.

There are limitations in the present study. (1) The sample size is small. Furthermore, this single and retrospective cohort of cases and controls may produce selection bias. To diminish the bias, we will perform pivotal evaluation of the diagnostic assay in a large cohort by using a prospective-specimen collection and retrospective-blinded evaluation design [40]. (2) A panel of five plasma miRNAs can diagnose SCC with 90% sensitivity and 94% specificity. However, a panel of four plasma miRNAs has only 67% sensitivity and 68% specificity for diagnosing NSCLC, which may not provide sufficient diagnostic significance in clinical settings. Cell-free circulating tumor DNA and DNA methylation status of gene promoters have been studied as potential liquid biopsy tests for lung cancer [41,42,43]. Our current endeavors are to compare and integrating plasma miRNA biomarkers with cell-free DNA biomarkers in order to enhance the early detection of lung cancer. (3) In this study, our primary objective was to evaluate the efficiency, speed, and broad measurement capabilities of the MiSeqDx method in analyzing miRNA expressions in plasma. We are conducting a new study that involves a direct comparison of the MiSeqDx system with PCR and WGS. This comparative analysis will utilize positive and negative control samples to evaluate the analytic performance of the MiSeqDx system for plasma miRNA analysis, including parameters such as limit of detection and specificity.

## 4. Materials and Methods

### 4.1. Participants and Plasma Preparation

The study protocol was approved by the Institutional Review Boards of the University of Maryland Baltimore. The surgical pathologic staging was determined according to the TNM classification of the International Union Against Cancer with the American Joint Committee on Cancer and the International Staging System for Lung Cancer [44]. Histopathologic classification was determined according to the World Health Organization classification [45]. Altogether, we recruited 39 patients with NSCLC and 32 cancer-free controls (Table 5). Blood samples were collected with the written informed consent from participants and obtained before therapeutic intervention using BD Vacutainer^®^ Venous Blood Collection Tubes (Becton, Dickinson and Company, Franklin Lakes, NJ, USA). The blood samples were immediately processed for plasma preparation via centrifugation at 3000 rpm (1900× *g*) for 10 min at 4 °C within less than 2 h after collection, as previously described in our published works [14,15,16,17,18,19,20,21].

### 4.2. RNA Isolation and cDNA Synthesis

Total RNA, including small RNA, was extracted from 200 µL of plasma using the miRNeasy Serum/Plasma kit (Qiagen, Hilden, Germany) following the manufacturer’s protocol. The resulting RNA was eluted in a final volume of 14 µL and stored at −70 °C until library construction.

### 4.3. MiRNA Library Construction, Quality Control, Sequencing, and RNAseq Data Analysis

The miRNA libraries were prepared using the QIAseq miRNA Library Kit (12) and QIAseq miRNA 12 Index IL (12) (QIAGEN, Aarhus, Denmark) according to the manufacturer’s protocol. Briefly, RNA was ligated with 3′ and 5′ adapters, followed by reverse transcription, cDNA amplification, and unique index sequence addition. Quality evaluation of the constructed libraries was performed using the Bioanalyzer with the High-Sensitivity DNA Analysis Assay (Agilent, Santa Clara, CA, USA), or by 8% TBE gel (Thermo Fisher Scientific, Santa Clara, CA, USA) electrophoresis. Quantification of the prepared libraries was performed using the Qubit™ 4 Fluorometer with Qubit^®^ dsDNA HS Assay Kits (Thermo Fisher Scientific). On the day of sequencing, four libraries were pooled in equimolar ratios and supplemented with 10% PhiX Control v3 (Illumina, San Diego, CA, USA). The sequencing libraries were then clustered at a final concentration of 15 pM in the flow cell and sequenced for 150 cycles using the MiSeq Reagent Kit v3 (150-cycle) on a MiSeq System (Illumina). Libraries were subjected to single-ended strand-specific sequencing with 75-bp-length reads, which resulted in approximately 4 to 7 million reads per sample. The Fast Quality Control (FASTQ) files of the sequenced samples were uploaded and analyzed using the Geneglobe RNAseq Analysis Portal 3.0 (QIAGEN) [46]. The sequenced libraries were mapped to the human genome (Genome Reference Consortium GRCh38) and annotated in reference to miRBase_v22, Homo sapiens (GRCh38.103). Secondary analysis of the Geneglobe RNAseq Analysis Portal 3.0 was powered via the Qiagen CLC Genomics Workbench and QIAGEN Ingenuity Pathway Analysis (IPA) (QIAGEN).

### 4.4. Quantitative Real-Time PCR (qRT-PCR) Analysis of Hemolysis in Plasma

To assess the hemolysis status of the sequenced plasma samples, we utilized a previously described miRNA-qRT-PCR-based method [25,47]. The expression levels of miR-23a-5p and miR-451a were detected using qRT-PCR. The miRNAs were reverse-transcribed to cDNA using the miRCURY LNA RT Kit (Qiagen) and diluted 1:20 in H_2_O for qRT-PCR quantification. The specific individual miRCURY LNA miRNA PCR Assays (Qiagen) and miRCURY^®^ LNA^®^ miRNA SYBR^®^ Green PCR Kit were used to determine the expression levels of miR-23a-3p and miR-451a in each sample. All qPCR reactions were performed in triplicate using the Bio-Rad CFX real-time PCR detection system (Bio-Rad, Hercules, CA, USA).

### 4.5. Statistical Analysis

ANOVA analysis was employed to determine whether significant differences in miRNA expression levels exist across various groups. The miRNAs were further analyzed using the least absolute shrinkage and selection operator (LASSO) to find the best combination of miRNAs to be used as a panel of diagnostic biomarkers. The panel of identified miRNAs was examined via Pearson’s correlation analysis to explore the presence of collinearity, which may reduce predictive accuracy. The diagnostic power of these sets of miRNAs was further explored using multiple variables binary logistic regression with ROC (Receiver Operating Characteristic) and AUC (area under the curve) to obtain the predicting algorithms, classification sensitivity, and specificity. Associations of miRNAs with age, smoking pack years, and cancer stages were analyzed using one-way ANOVA. Sex and race were compared using a chi-squared test.

## 5. Conclusions

Our study highlights that the MiSeqDx system can measure miRNA expression in plasma in an efficient, rapid, and comprehensive manner. Furthermore, we have developed three panels of plasma miRNA biomarkers that can aid in the early detection and histological classification of NSCLC. However, additional technology standardization and a large, prospective study are necessary for validating the biomarkers and establishing their clinical utility.

## Figures and Tables

**Table 1:** The differentially expressed miRNAs between patients with NSCLC and controls.

miRNAs	FC	*p*-Value
miR-1246	2.32	3.62 × 10^−4^
miR-129-5p	8.93	1.34 × 10^−7^
miR-1299	−3.19	3.06 × 10^−4^
miR-205-5p	2.55	2.30 × 10^−4^
miR-215-5p	−2.33	2.64 × 10^−5^
miR-31-5p	−4.17	8.08 × 10^−5^
miR-483-3p	−5.2	5.28 × 10^−9^
miR-483-5p	−3	2.56 × 10^−5^

FC, fold change.

**Table 2:** The differentially expressed miRNAs between patients with SCC and controls.

miRNAs	FC	*p*-Value
miR-1299	−7.37	1.55 × 10^−7^
miR-141-3p	−2.39	1.27 × 10^−3^
miR-200a-3p	−4.1	2.66 × 10^−6^
miR-200b-3p	−2.95	4.52 × 10^−4^
miR-200b-5p	−3.81	9.06 × 10^−4^
miR-205-5p	3.69	8.16 × 10^−6^
miR-215-5p	−2.75	2.99 × 10^−5^
miR-31-5p	−6.44	3.85 × 10^−5^
miR-429	−3.47	1.69 × 10^−4^
miR-483-3p	−6.03	1.08 × 10^−7^
miR-483-5p	−3.68	2.89 × 10^−5^

FC, fold change.

**Table 3:** The differentially expressed miRNAs between patients with AC and controls.

miRNAs	FC	*p*-Value
miR-105-5p	15.79	4.93 × 10^−5^
miR-129-2-3p	8.79	7.12 × 10^−5^
miR-129-5p	17.4	3.82 × 10^−11^
miR-483-3p	−4.54	1.05 × 10^−5^

FC, fold change.

**Table 4:** The differentially expressed miRNAs between patients with AC and SCC.

Name	FC	*p*-Value
miR-105-5p	18.95	2.18 × 10^−4^
miR-129-2-3p	7.72	4.96 × 10^−4^
miR-129-5p	21.39	2.27 × 10^−10^
miR-141-3p	4.1	3.08 × 10^−6^
miR-200a-3p	3.36	3.14 × 10^−4^
miR-200b-3p	3.63	1.66 × 10^−4^
miR-200c-3p	2.64	5.66 × 10^−5^
miR-218-5p	5.9	1.52 × 10^−4^
miR-375-3p	4.29	2.04 × 10^−6^

FC, fold change.

**Table 5:** Characteristics of the cohort of NSCLC patients and cancer-free smokers.

	AC Cases (*n* = 19)	SCC (*n* = 20)	Controls (*n* = 32)	*p*-Value
Age	65.5 (SD, 7.8)	67.0 (SD, 6.4)	60.9 (SD, 11.2)	0.0877
Sex				0.2872
Female	9	5	14	
Male	10	15	18	
Race				0.8261
African Americans	6	7	12	
White Americans	13	13	19	
Hispanic	0	0	1	
Smoking pack years (median)	30	50	14	0.0051
Stage				
Stage I	2	3		
Stage II	1	1		
Stage III	5	2		
Stage IV	1	3		
Unknown	10	11		
Histological type				

NSCLC, non-small cell lung cancer; AC, adenocarcinoma; SCC, squamous cell cancer; SD, standard deviation. Age and smoking pack years of cases and control were compared via one-way ANOVA. Sex and race were compared via chi-squared test.

## Data Availability

The data that support the findings of this study are available from the corresponding author upon a reasonable request.

## Supplementary Materials

The supporting information can be downloaded at: https://www.mdpi.com/article/10.3390/ijms241210277/s1.
