# Supplementary material for: Utilizing MiSeq Sequencing to Detect Circulating microRNAs in Plasma for Improved Lung Cancer Diagnosis
Source: Int J Mol Sci. 2023 Jun 17;24(12):10277. doi: 10.3390/ijms241210277 (PMC10299334; doi:10.3390/ijms241210277)
Supplement: Supplementary file 1 [file ijms-24-10277-s001.zip › ijms-2432524-supplementary.pdf]

Supplementary files

Supplemental table S1: Quality control summary- Reads quality of all the samples

Sample name	Reads	UMI reads	Avg Q score, UMI reads	UMI reads annotated	UMI reads annotated with miRBase
PlasmaVA686B_S1	5,260,354	807,361	55.17	463,565 (57.43%)	443,073 (54.89%)
PlasmaUM713B_S2	6,656,545	2,243,882	41.36	1,118,115 (49.83%)	1,030,540 (45.93%)
PlasmaVA516SCC_S2	5,755,835	2,047,557	44.96	1,067,977 (52.16%)	1,014,378 (49.55%)
UM773AC_S2	4,558,980	894,947	51.52	630,609 (70.48%)	598,516 (66.89%)
UM778AC-IDP07_S3	8,154,563	939,008	52.61	396,702 (42.25%)	387,834 (41.31%)
PlasmaVA189AC_S1	6,389,037	1,209,276	54.57	967,397 (80.01%)	943,687 (78.04%)
UMs753SCC-IDP3_S1	5,208,597	1,672,091	45.42	819,905 (49.04%)	795,055 (47.56%)
UM754mAC-IDP4_S2	4,384,680	1,003,951	48.19	604,062 (60.17%)	591,766 (58.95%)
PlasmaUM711SCC-IDP2_S1	6,010,282	909,432	54.91	661,426 (72.73%)	603,620 (66.38%)
PlasmaUM736AC_S2	5,386,123	1,578,289	44.85	1,148,819 (72.80%)	949,779 (60.19%)
PlasmaUM692B_S1	5,954,668	2,428,421	46.17	2,075,713 (85.50%)	1,895,158 (78.06%)
PlasmaUM636SCC_S4	5,741,784	3,274,957	41.27	2,870,619 (87.69%)	2,630,572 (80.36%)
PlasmaUM705mAC_S3	6,474,633	1,080,689	51.9	679,258 (62.86%)	629,270 (58.24%)
PlasmaUM764B-IDP10_S3	5,267,368	623,563	56.45	477,496 (76.58%)	467,585 (74.99%)
PlasmaUM652B_S3	4,548,156	434,769	57.39	205,973 (47.38%)	183,607 (42.23%)
PlasmaUM686AC_S4	5,853,101	2,500,558	44.02	2,218,644 (88.75%)	2,082,164 (83.29%)
UM701-2AC-IDP7_S1	8,476,726	818,979	55.8	551,080 (67.29%)	538,818 (65.80%)
PlasmaUM735AC_S3	6,091,752	2,924,606	40.99	2,230,443 (76.27%)	1,969,870 (67.36%)
PlasmaUM656B_S4	6,790,054	856,474	56.77	397,266 (46.39%)	379,001 (44.25%)
PlasmaUM785B-IDP11_S4	5,807,056	582,308	56.86	454,804 (78.11%)	435,954 (74.87%)
PlasmaUM547B_S2	4,597,134	1,093,309	48.82	209,372 (19.15%)	195,717 (17.90%)
UM726B_S2	7,589,584	858,280	54.42	455,967 (53.13%)	430,805 (50.20%)
PlasmaUM662B_S4	8,262,025	724,116	57.78	387,393 (53.50%)	373,376 (51.56%)
UM774B_S3	6,099,959	902,232	53.13	627,317 (69.54%)	611,067 (67.74%)
PlasmaUM635SCC_S3	6,351,655	1,886,986	44.28	1,445,462 (76.62%)	1,333,649 (70.70%)
UMs709AC-IDP10_S1	6,966,012	686,093	57.27	338,827 (49.39%)	320,520 (46.72%)
PlasmaUM693B_S1	5,465,967	2,781,213	43.3	2,437,623 (87.69%)	2,085,236 (75.02%)
VA700B_S1	7,013,968	1,377,193	49.82	844,274 (61.31%)	731,733 (53.14%)
PlasmaUM690B_S2	6,596,070	1,564,653	52.06	1,220,428 (78.01%)	1,158,474 (74.05%)
PlasmaUM645B_S1	4,627,280	1,791,823	44.73	1,456,638 (81.31%)	1,283,514 (71.64%)
PlasmaUM748AC_S4	5,599,128	2,301,565	43.85	1,982,709 (86.18%)	1,855,694 (80.66%)
UM680SCC-IDP01_S1	7,132,387	1,416,383	47.78	805,107 (56.85%)	766,096 (54.09%)
UM751SCC-IDP09_S3	6,455,923	1,433,551	50.1	903,036 (63%)	868,586 (60.60%)
PlasmaUM727B_S2	5,698,657	2,847,524	42.54	2,391,856 (84.03%)	1,985,762 (69.76%)
PlasmaUM715B-IDP4_S2	6,598,054	2,130,191	51.41	1,771,627 (83.18%)	1,579,302 (74.15%)

PlasmaUM719B_S2	5,475,318	2,428,321	43.91	2,019,080 (83.18%)	1,678,275 (69.14%)
PlasmaUM777B_S4	4,820,785	1,051,590	52.14	691,396 (65.76%)	681,075 (64.78%)
PlasmaUM708AC_S3	7,048,518	2,642,830	46.11	2,223,966 (84.21%)	1,655,873 (62.70%)
UMs757B-IDP5_S2	6,547,329	978,466	56.37	649,742 (66.41%)	618,494 (63.22%)
PlasmaUM717AC_S1	7,673,975	1,770,220	48.79	1,107,019 (62.55%)	1,083,231 (61.20%)
PlasmaUM633B_S3	8,164,992	882,020	57.3	458,808 (52.02%)	444,119 (50.36%)
PlasmaUM786SCC_S5	5,551,233	616,112	56.27	396,702 (64.40%)	386,713 (62.77%)
PlasmaUM674AC_S1	5,898,851	667,442	56.09	478,917 (71.76%)	466,721 (69.93%)
UM759B-IDP12_S2	7,699,216	2,061,477	48.06	1,421,543 (68.97%)	1,390,874 (67.48%)
PlasmaUM658SCC_S3	6,841,276	5,144,647	38.28	4,713,021 (91.66%)	4,145,097 (80.61%)
UM741B_S4	8,038,925	1,124,084	53.4	688,693 (61.27%)	656,539 (58.41%)
PlasmaUM676B_S3	4,295,469	678,167	55.88	469,791 (69.28%)	462,424 (68.20%)
PlasmaUM642SCC_S4	5,977,042	2,433,505	42.92	1,952,169 (80.24%)	1,638,716 (67.36%)
UMs766AC-IDP8_S3	6,739,436	941,325	57.1	620,515 (65.92%)	595,086 (63.22%)
UMs761SCC-IDP7_S3	6,261,816	1,445,795	53.75	932,185 (64.49%)	822,003 (56.87%)
PlasmaUM667SCC_S4	6,040,219	2,709,822	41.51	2,108,957 (77.86%)	1,828,245 (67.50%)
PlasmaUM373B_S4	6,015,391	931,031	54.72	677,735 (72.80%)	664,368 (71.36%)
PlasmaUM696SCC-IDP1_S1	6,088,704	1,006,620	54.43	773,325 (76.84%)	710,985 (70.64%)
UM760B-IDP02_S1	7,872,949	2,614,748	48	2,122,653 (81.20%)	2,071,001 (79.23%)
PlasmaUM385B_S4	5,643,075	1,140,624	53.45	920,039 (80.67%)	891,634 (78.18%)
PlasmaUM641B_S1	5,754,477	3,017,242	41.88	2,739,038 (90.82%)	2,593,720 (86.01%)
PlasmaUM720SCC_S3	5,685,516	1,930,624	47.54	1,590,274 (82.39%)	1,290,894 (66.88%)
PlasmaUM730AC_S2	5,825,552	3,462,730	39.48	3,004,193 (86.79%)	2,628,896 (75.95%)
PlasmaUM737AC_S2	6,056,096	3,187,916	39.98	2,553,435 (80.12%)	2,410,971 (75.65%)
PlasmaUM734SCC-IDP7_S4	5,445,338	652,449	55.16	430,365 (65.97%)	409,768 (62.81%)
PlasmaUM644B_S1	5,505,061	3,626,951	40.4	3,264,500 (90.05%)	2,881,601 (79.49%)
PlasmaUM745SCC-IDP9_S2	6,090,589	747,149	55.42	506,134 (67.75%)	490,949 (65.72%)
PlasmaUM728SCC-IDP6_S3	6,014,862	2,326,986	49.67	2,077,180 (89.28%)	1,976,822 (84.97%)
UM740B_S3	8,653,435	962,727	55.3	547,615 (56.89%)	536,532 (55.73%)
PlasmaUM742B_S2	5,791,170	3,242,083	41.03	2,890,938 (89.21%)	2,488,589 (76.79%)
PlasmaVA525SCC_S4	6,291,716	736,579	56.35	223,683 (30.37%)	215,010 (29.19%)
PlasmaVA530SCC_S1	6,160,632	761,928	55.91	217,405 (28.54%)	210,569 (27.64%)
PlasmaUM659AC_S3	5,943,502	2,954,833	43.24	2,694,674 (91.24%)	2,522,405 (85.40%)
PlasmaVA473SCC_S1	6,226,269	1,815,741	44.64	827,024 (45.55%)	762,215 (41.98%)
PlasmaUM661AC_S4	5,306,869	1,147,112	48.53	780,368 (68.03%)	653,157 (56.94%)
PlasmaUM767B_S2	6,407,293	433,067	57.06	217,837 (50.30%)	212,486 (49.07%)

Supplemental table S2: Creation of UMI reads from raw reads

Sample name	Input reads	Avg Q score, input reads	Discarded reads	UMI groups	Merged UMI groups	Avg Q score, UMI reads	Avg reads per UMI	UMIs with less than 9 reads
PlasmaVA686B_S1	5,260,354	36.45	2,279,350	956,134	807,361	55.17	3	96.62%
PlasmaUM713B_S2	6,656,545	36.49	3,767,799	2,309,253	2,243,882	41.36	1	100.00%
PlasmaVA516SCC_S2	5,755,835	36.53	2,598,540	2,124,718	2,047,557	44.96	1	100.00%
UM773AC_S2	4,558,980	36.37	2,417,462	965,005	894,947	51.52	2	99.86%
UM778AC-IDP07_S3	8,154,563	35.75	5,449,249	1,066,746	939,008	52.61	2	99.25%
PlasmaVA189AC_S1	6,389,037	36.52	2,348,806	1,361,519	1,209,276	54.57	3	98.18%
UMs753SCC-IDP3_S1	5,208,597	36.3	2,481,300	1,785,357	1,672,091	45.42	1	99.98%
UM754mAC-IDP4_S2	4,384,680	36.47	2,506,577	1,065,447	1,003,951	48.19	1	99.98%
PlasmaUM711SCC-IDP2_S1	6,010,282	36.35	2,734,678	1,034,103	909,432	54.91	3	96.87%
PlasmaUM736AC_S2	5,386,123	36.44	2,936,797	1,663,968	1,578,289	44.85	1	99.99%
PlasmaUM692B_S1	5,954,668	36.48	1,846,070	2,620,865	2,428,421	46.17	1	99.99%
PlasmaUM636SCC_S4	5,741,784	36.44	1,300,709	3,636,519	3,274,957	41.27	1	99.92%
PlasmaUM705mAC_S3	6,474,633	36.46	3,756,693	1,210,453	1,080,689	51.9	2	99.77%
PlasmaUM764B-IDP10_S3	5,267,368	30.99	2,205,674	816,248	623,563	56.45	4	87.44%
PlasmaUM652B_S3	4,548,156	36.36	1,742,159	538,597	434,769	57.39	6	70.89%
PlasmaUM686AC_S4	5,853,101	36.41	1,977,141	2,808,841	2,500,558	44.02	1	99.94%
UM701-2AC-IDP7_S1	8,476,726	36.06	5,029,998	1,030,602	818,979	55.8	4	93.89%
PlasmaUM735AC_S3	6,091,752	36.54	2,393,126	3,025,534	2,924,606	40.99	1	99.99%
PlasmaUM656B_S4	6,790,054	36.32	2,939,052	1,022,860	856,474	56.77	4	93.03%
PlasmaUM785B-IDP11_S4	5,807,056	31.69	2,304,948	789,884	582,308	56.86	6	75.14%
PlasmaUM547B_S2	4,597,134	36.4	2,451,507	1,139,689	1,093,309	48.82	1	99.98%
UM726B_S2	7,589,584	35.95	4,792,752	963,282	858,280	54.42	3	98.60%
PlasmaUM662B_S4	8,262,025	36.4	3,209,251	902,888	724,116	57.78	6	65.72%
UM774B_S3	6,099,959	36.38	3,646,389	997,020	902,232	53.13	2	99.62%
PlasmaUM635SCC_S3	6,351,655	36.43	3,465,962	2,034,686	1,886,986	44.28	1	99.98%
UMs709AC-IDP10_S1	6,966,012	36.1	2,735,535	850,662	686,093	57.27	6	74.67%
PlasmaUM693B_S1	5,465,967	36.53	1,325,059	3,117,789	2,781,213	43.3	1	99.95%
VA700B_S1	7,013,968	35.93	4,032,817	1,476,918	1,377,193	49.82	2	99.94%
PlasmaUM690B_S2	6,596,070	36.45	2,615,344	1,828,850	1,564,653	52.06	2	99.68%
PlasmaUM645B_S1	4,627,280	36.46	1,801,101	1,971,654	1,791,823	44.73	1	99.96%
PlasmaUM748AC_S4	5,599,128	36.52	2,005,887	2,662,784	2,301,565	43.85	1	99.90%
UM680SCC-IDP01_S1	7,132,387	36.07	4,457,117	1,513,119	1,416,383	47.78	1	99.98%
UM751SCC-IDP09_S3	6,455,923	36.05	3,295,531	1,596,176	1,433,551	50.1	2	99.91%
PlasmaUM727B_S2	5,698,657	36.51	1,681,951	3,100,812	2,847,524	42.54	1	99.97%

PlasmaUM715B-IDP4_S2	6,598,054	36.36	1,491,833	2,403,215	2,130,191	51.41	2	99.81%
PlasmaUM719B_S2	5,475,318	36.44	1,824,514	2,630,752	2,428,321	43.91	1	99.98%
PlasmaUM777B_S4	4,820,785	36.45	2,135,736	1,216,969	1,051,590	52.14	2	99.69%
PlasmaUM708AC_S3	7,048,518	36.45	2,574,094	2,894,797	2,642,830	46.11	1	99.98%
UMs757B-IDP5_S2	6,547,329	36.37	2,321,795	1,184,745	978,466	56.37	4	93.43%
PlasmaUM717AC_S1	7,673,975	35.18	3,776,345	2,082,612	1,770,220	48.79	2	99.85%
PlasmaUM633B_S3	8,164,992	36.4	3,540,393	1,084,193	882,020	57.3	5	86.28%
PlasmaUM786SCC_S5	5,551,233	36.44	2,767,034	743,039	616,112	56.27	4	91.15%
PlasmaUM674AC_S1	5,898,851	36.45	3,079,502	808,219	667,442	56.09	4	94.05%
UM759B-IDP12_S2	7,699,216	36.08	3,724,771	2,270,784	2,061,477	48.06	1	99.96%
PlasmaUM658SCC_S3	6,841,276	36.51	1,068,864	5,336,122	5,144,647	38.28	1	99.98%
UM741B_S4	8,038,925	35.93	4,774,214	1,258,105	1,124,084	53.4	2	99.36%
PlasmaUM676B_S3	4,295,469	36.39	1,602,893	841,004	678,167	55.88	3	95.20%
PlasmaUM642SCC_S4	5,977,042	36.47	2,486,809	2,636,792	2,433,505	42.92	1	99.97%
UMs766AC-IDP8_S3	6,739,436	36.1	1,681,215	1,147,766	941,325	57.1	5	83.62%
UMs761SCC-IDP7_S3	6,261,816	36.37	1,940,667	1,652,521	1,445,795	53.75	2	99.16%
PlasmaUM667SCC_S4	6,040,219	36.46	2,500,493	2,813,770	2,709,822	41.51	1	99.99%
PlasmaUM373B_S4	6,015,391	36.46	2,333,984	1,086,471	931,031	54.72	3	92.67%
PlasmaUM696SCC-IDP1_S1	6,088,704	32.57	2,664,292	1,224,793	1,006,620	54.43	3	97.83%
UM760B-IDP02_S1	7,872,949	35.76	2,514,728	3,165,780	2,614,748	48	2	99.79%
PlasmaUM385B_S4	5,643,075	36.44	1,523,447	1,316,908	1,140,624	53.45	3	95.49%
PlasmaUM641B_S1	5,754,477	36.42	1,448,161	3,458,536	3,017,242	41.88	1	99.88%
PlasmaUM720SCC_S3	5,685,516	36.55	2,180,646	2,101,315	1,930,624	47.54	1	99.98%
PlasmaUM730AC_S2	5,825,552	36.47	1,702,314	3,602,757	3,462,730	39.48	1	99.98%
PlasmaUM737AC_S2	6,056,096	36.37	2,064,705	3,422,590	3,187,916	39.98	1	99.96%
PlasmaUM734SCC-IDP7_S4	5,445,338	36.34	2,976,734	749,029	652,449	55.16	3	95.77%
PlasmaUM644B_S1	5,505,061	36.48	970,062	3,812,966	3,626,951	40.4	1	99.99%
PlasmaUM745SCC-IDP9_S2	6,090,589	31.88	3,151,428	925,645	747,149	55.42	3	95.11%
PlasmaUM728SCC-IDP6_S3	6,014,862	36.36	1,062,915	2,734,097	2,326,986	49.67	2	99.85%
UM740B_S3	8,653,435	35.93	5,161,326	1,108,504	962,727	55.3	3	97.51%
PlasmaUM742B_S2	5,791,170	36.56	1,523,626	3,529,658	3,242,083	41.03	1	99.96%
PlasmaVA525SCC_S4	6,291,716	36.45	2,909,851	892,188	736,579	56.35	4	91.18%
PlasmaVA530SCC_S1	6,160,632	36.38	2,963,293	865,069	761,928	55.91	4	93.70%
PlasmaUM659AC_S3	5,943,502	36.55	1,462,260	3,417,533	2,954,833	43.24	1	99.90%
PlasmaVA473SCC_S1	6,226,269	36.55	3,480,724	1,871,387	1,815,741	44.64	1	100.00%
PlasmaUM661AC_S4	5,306,869	36.49	3,101,064	1,233,985	1,147,112	48.53	1	99.98%
PlasmaUM767B_S2	6,407,293	36.45	3,779,346	521,144	433,067	57.06	6	75.24%

Supplemental table S3: miRNA found in samples

	miRBase (Homo sapiens)
Records in source	1,917
Records found in sample	-
PlasmaVA686B_S1	571 (29.79%)
PlasmaUM713B_S2	753 (39.28%)
PlasmaVA516SCC_S2	747 (38.97%)
UM773AC_S2	706 (36.83%)
UM778AC-IDP07_S3	532 (27.75%)
PlasmaVA189AC_S1	738 (38.50%)
UMs753SCC-IDP3_S1	719 (37.51%)
UM754mAC-IDP4_S2	582 (30.36%)
PlasmaUM711SCC-IDP2_S1	707 (36.88%)
PlasmaUM736AC_S2	773 (40.32%)
PlasmaUM692B_S1	893 (46.58%)
PlasmaUM636SCC_S4	889 (46.37%)
PlasmaUM705mAC_S3	606 (31.61%)
PlasmaUM764B-IDP10_S3	536 (27.96%)
PlasmaUM652B_S3	535 (27.91%)
PlasmaUM686AC_S4	856 (44.65%)
UM701-2AC-IDP7_S1	623 (32.50%)
PlasmaUM735AC_S3	915 (47.73%)
PlasmaUM656B_S4	531 (27.70%)
PlasmaUM785B-IDP11_S4	575 (29.99%)
PlasmaUM547B_S2	487 (25.40%)
UM726B_S2	575 (29.99%)
PlasmaUM662B_S4	519 (27.07%)
UM774B_S3	586 (30.57%)
PlasmaUM635SCC_S3	834 (43.51%)
UMs709AC-IDP10_S1	562 (29.32%)
PlasmaUM693B_S1	851 (44.39%)
VA700B_S1	719 (37.51%)
PlasmaUM690B_S2	669 (34.90%)
PlasmaUM645B_S1	761 (39.70%)
PlasmaUM748AC_S4	807 (42.10%)
UM680SCC-IDP01_S1	768 (40.06%)
UM751SCC-IDP09_S3	682 (35.58%)
PlasmaUM727B_S2	863 (45.02%)
PlasmaUM715B-IDP4_S2	837 (43.66%)
PlasmaUM719B_S2	866 (45.17%)
PlasmaUM777B_S4	530 (27.65%)
PlasmaUM708AC_S3	893 (46.58%)
UMs757B-IDP5_S2	615 (32.08%)

PlasmaUM717AC_S1	673 (35.11%)
PlasmaUM633B_S3	529 (27.60%)
PlasmaUM786SCC_S5	529 (27.60%)
PlasmaUM674AC_S1	531 (27.70%)
UM759B-IDP12_S2	777 (40.53%)
PlasmaUM658SCC_S3	965 (50.34%)
UM741B_S4	619 (32.29%)
PlasmaUM676B_S3	490 (25.56%)
PlasmaUM642SCC_S4	854 (44.55%)
UMs766AC-IDP8_S3	570 (29.73%)
UMs761SCC-IDP7_S3	712 (37.14%)
PlasmaUM667SCC_S4	876 (45.70%)
PlasmaUM373B_S4	571 (29.79%)
PlasmaUM696SCC-IDP1_S1	694 (36.20%)
UM760B-IDP02_S1	853 (44.50%)
PlasmaUM385B_S4	659 (34.38%)
PlasmaUM641B_S1	841 (43.87%)
PlasmaUM720SCC_S3	809 (42.20%)
PlasmaUM730AC_S2	953 (49.71%)
PlasmaUM737AC_S2	915 (47.73%)
PlasmaUM734SCC-IDP7_S4	565 (29.47%)
PlasmaUM644B_S1	925 (48.25%)
PlasmaUM745SCC-IDP9_S2	552 (28.79%)
PlasmaUM728SCC-IDP6_S3	820 (42.78%)
UM740B_S3	507 (26.45%)
PlasmaUM742B_S2	914 (47.68%)
PlasmaVA525SCC_S4	437 (22.80%)
PlasmaVA530SCC_S1	486 (25.35%)
PlasmaUM659AC_S3	864 (45.07%)
PlasmaVA473SCC_S1	733 (38.24%)
PlasmaUM661AC_S4	675 (35.21%)
PlasmaUM767B_S2	422 (22.01%)

Supplemental table S4. Unique search sequences

Sample name	Annotated (of total)	Annotated with miRBase	Unannotated (of total)	Total (of total)
PlasmaVA686B_S1	8,091 (4.17%)	5,939 (3.06%)	186,065 (95.83%)	194,156 (100%)
PlasmaUM713B_S2	18,986 (3.85%)	14,263 (2.90%)	473,571 (96.15%)	492,557 (100%)
PlasmaVA516SCC_S2	18,266 (4.86%)	14,356 (3.82%)	357,463 (95.14%)	375,729 (100%)
UM773AC_S2	12,061 (8.39%)	9,627 (6.70%)	131,627 (91.61%)	143,688 (100%)
UM778AC-IDP07_S3	8,342 (2.41%)	5,867 (1.69%)	337,848 (97.59%)	346,190 (100%)
PlasmaVA189AC_S1	14,591 (13.08%)	12,413 (11.13%)	96,972 (86.92%)	111,563 (100%)
UMs753SCC-IDP3_S1	14,453 (2.65%)	10,279 (1.89%)	530,233 (97.35%)	544,686 (100%)
UM754mAC-IDP4_S2	9,461 (3.81%)	7,274 (2.93%)	238,943 (96.19%)	248,404 (100%)
PlasmaUM711SCC-IDP2_S1	12,683 (9.08%)	10,125 (7.25%)	127,052 (90.92%)	139,735 (100%)
PlasmaUM736AC_S2	20,298 (7.52%)	15,513 (5.74%)	249,776 (92.48%)	270,074 (100%)
PlasmaUM692B_S1	29,657 (15.02%)	24,141 (12.23%)	167,732 (84.98%)	197,389 (100%)
PlasmaUM636SCC_S4	31,530 (15.15%)	25,255 (12.13%)	176,613 (84.85%)	208,143 (100%)
PlasmaUM705mAC_S3	12,576 (6.07%)	9,382 (4.53%)	194,642 (93.93%)	207,218 (100%)
PlasmaUM764B-IDP10_S3	7,589 (8.65%)	6,106 (6.96%)	80,144 (91.35%)	87,733 (100%)
PlasmaUM652B_S3	7,018 (4.79%)	4,942 (3.38%)	139,353 (95.21%)	146,371 (100%)
PlasmaUM686AC_S4	24,884 (13.87%)	20,872 (11.63%)	154,589 (86.13%)	179,473 (100%)
UM701-2AC-IDP7_S1	10,725 (7.40%)	8,648 (5.97%)	134,142 (92.60%)	144,867 (100%)
PlasmaUM735AC_S3	31,180 (7.67%)	24,220 (5.96%)	375,366 (92.33%)	406,546 (100%)
PlasmaUM656B_S4	8,198 (2.84%)	5,803 (2.01%)	280,090 (97.16%)	288,288 (100%)
PlasmaUM785B-IDP11_S4	8,788 (12.21%)	7,343 (10.20%)	63,212 (87.79%)	72,000 (100%)
PlasmaUM547B_S2	7,189 (1.66%)	4,686 (1.08%)	424,889 (98.34%)	432,078 (100%)
UM726B_S2	9,745 (3.96%)	6,926 (2.81%)	236,650 (96.04%)	246,395 (100%)
PlasmaUM662B_S4	7,527 (3.48%)	5,549 (2.57%)	208,631 (96.52%)	216,158 (100%)
UM774B_S3	9,053 (5.75%)	7,095 (4.51%)	148,278 (94.25%)	157,331 (100%)
PlasmaUM635SCC_S3	21,759 (8.06%)	16,926 (6.27%)	248,037 (91.94%)	269,796 (100%)
UMs709AC-IDP10_S1	8,644 (4.53%)	5,923 (3.10%)	182,165 (95.47%)	190,809 (100%)
PlasmaUM693B_S1	26,211 (14.35%)	20,913 (11.45%)	156,403 (85.65%)	182,614 (100%)
VA700B_S1	15,996 (4.50%)	11,954 (3.36%)	339,599 (95.50%)	355,595 (100%)
PlasmaUM690B_S2	14,978 (7.80%)	11,704 (6.10%)	176,998 (92.20%)	191,976 (100%)
PlasmaUM645B_S1	20,351 (9.60%)	16,391 (7.73%)	191,584 (90.40%)	211,935 (100%)
PlasmaUM748AC_S4	21,512 (10.28%)	17,417 (8.33%)	187,659 (89.72%)	209,171 (100%)
UM680SCC-IDP01_S1	14,743 (3.93%)	11,533 (3.08%)	360,099 (96.07%)	374,842 (100%)
UM751SCC-IDP09_S3	12,242 (3.69%)	9,342 (2.82%)	319,457 (96.31%)	331,699 (100%)
PlasmaUM727B_S2	27,240 (11.78%)	21,196 (9.17%)	204,011 (88.22%)	231,251 (100%)
PlasmaUM715B-IDP4_S2	21,701 (13.89%)	17,009 (10.89%)	134,504 (86.11%)	156,205 (100%)
PlasmaUM719B_S2	26,597 (12%)	21,112 (9.52%)	195,076 (88%)	221,673 (100%)
PlasmaUM777B_S4	8,337 (3.99%)	6,049 (2.89%)	200,836 (96.01%)	209,173 (100%)
PlasmaUM708AC_S3	28,525 (12.30%)	22,374 (9.65%)	203,325 (87.70%)	231,850 (100%)

UMs757B-IDP5_S2	10,025 (5.49%)	7,491 (4.10%)	172,542 (94.51%)	182,567 (100%)
PlasmaUM717AC_S1	14,628 (3.77%)	11,115 (2.86%)	373,789 (96.23%)	388,417 (100%)
PlasmaUM633B_S3	8,436 (3.10%)	5,795 (2.13%)	263,462 (96.90%)	271,898 (100%)
PlasmaUM786SCC_S5	6,769 (5.91%)	5,215 (4.55%)	107,764 (94.09%)	114,533 (100%)
PlasmaUM674AC_S1	7,847 (8.02%)	6,233 (6.37%)	89,938 (91.98%)	97,785 (100%)
UM759B-IDP12_S2	18,284 (4.68%)	14,532 (3.72%)	372,143 (95.32%)	390,427 (100%)
PlasmaUM658SCC_S3	41,392 (18.23%)	34,590 (15.23%)	185,710 (81.77%)	227,102 (100%)
UM741B_S4	10,946 (4.26%)	8,213 (3.20%)	246,102 (95.74%)	257,048 (100%)
PlasmaUM676B_S3	6,228 (5.82%)	4,738 (4.42%)	100,857 (94.18%)	107,085 (100%)
PlasmaUM642SCC_S4	25,079 (8.73%)	19,806 (6.90%)	262,045 (91.27%)	287,124 (100%)
UMs766AC-IDP8_S3	9,147 (5.07%)	6,858 (3.80%)	171,135 (94.93%)	180,282 (100%)
UMs761SCC-IDP7_S3	15,022 (5.20%)	11,065 (3.83%)	274,005 (94.80%)	289,027 (100%)
PlasmaUM667SCC_S4	31,446 (9.29%)	25,026 (7.39%)	307,128 (90.71%)	338,574 (100%)
PlasmaUM373B_S4	9,307 (8.29%)	7,373 (6.57%)	102,972 (91.71%)	112,279 (100%)
PlasmaUM696SCC-IDP1_S1	13,459 (9.38%)	10,763 (7.50%)	130,050 (90.62%)	143,509 (100%)
UM760B-IDP02_S1	22,430 (7.19%)	18,575 (5.96%)	289,375 (92.81%)	311,805 (100%)
PlasmaUM385B_S4	11,840 (11.34%)	9,654 (9.24%)	92,605 (88.66%)	104,445 (100%)
PlasmaUM641B_S1	25,667 (14.52%)	21,383 (12.10%)	151,114 (85.48%)	176,781 (100%)
PlasmaUM720SCC_S3	23,855 (12.47%)	19,148 (10.01%)	167,388 (87.53%)	191,243 (100%)
PlasmaUM730AC_S2	35,955 (13.62%)	29,832 (11.30%)	228,110 (86.38%)	264,065 (100%)
PlasmaUM737AC_S2	29,666 (10.13%)	23,753 (8.11%)	263,130 (89.87%)	292,796 (100%)
PlasmaUM734SCC-IDP7_S4	8,295 (6.15%)	6,470 (4.80%)	126,478 (93.85%)	134,773 (100%)
PlasmaUM644B_S1	38,344 (20.98%)	32,269 (17.65%)	144,442 (79.02%)	182,786 (100%)
PlasmaUM745SCC-IDP9_S2	8,936 (5.92%)	6,687 (4.43%)	142,016 (94.08%)	150,952 (100%)
PlasmaUM728SCC-IDP6_S3	20,678 (15.45%)	17,154 (12.82%)	113,136 (84.55%)	133,814 (100%)
UM740B_S3	7,653 (2.96%)	5,649 (2.18%)	250,906 (97.04%)	258,559 (100%)
PlasmaUM742B_S2	29,890 (14.74%)	24,527 (12.10%)	172,848 (85.26%)	202,738 (100%)
PlasmaVA525SCC_S4	6,193 (2.02%)	4,111 (1.34%)	301,013 (97.98%)	307,206 (100%)
PlasmaVA530SCC_S1	6,126 (2.01%)	4,281 (1.41%)	298,565 (97.99%)	304,691 (100%)
PlasmaUM659AC_S3	24,999 (17.49%)	20,872 (14.60%)	117,913 (82.51%)	142,912 (100%)
PlasmaVA473SCC_S1	17,418 (4.36%)	13,205 (3.30%)	382,135 (95.64%)	399,553 (100%)
PlasmaUM661AC_S4	15,760 (7.36%)	11,586 (5.41%)	198,401 (92.64%)	214,161 (100%)
PlasmaUM767B_S2	4,993 (4.48%)	3,727 (3.35%)	106,421 (95.52%)	111,414 (100%)

Supplemental table S5. The number of reads annotated with miRBase

Sample name	Annotated	Annotated	Unannotated	Total
PlasmaVA686B_S1	463,565 (57.43%)	443,073 (54.89%)	343,666 (42.57%)	807,231 (100%)
PlasmaUM713B_S2	1,118,115 (49.83%)	1,030,540 (45.93%)	1,125,632 (50.17%)	2,243,747 (100%)
PlasmaVA516SCC_S2	1,067,977 (52.16%)	1,014,378 (49.55%)	979,388 (47.84%)	2,047,365 (100%)
UM773AC_S2	630,609 (70.48%)	598,516 (66.89%)	264,120 (29.52%)	894,729 (100%)
UM778AC-IDP07_S3	396,702 (42.25%)	387,834 (41.31%)	542,222 (57.75%)	938,924 (100%)
PlasmaVA189AC_S1	967,397 (80.01%)	943,687 (78.04%)	241,766 (19.99%)	1,209,163 (100%)
UMs753SCC-IDP3_S1	819,905 (49.04%)	795,055 (47.56%)	851,905 (50.96%)	1,671,810 (100%)
UM754mAC-IDP4_S2	604,062 (60.17%)	591,766 (58.95%)	399,813 (39.83%)	1,003,875 (100%)
PlasmaUM711SCC-IDP2_S1	661,426 (72.73%)	603,620 (66.38%)	247,954 (27.27%)	909,380 (100%)
PlasmaUM736AC_S2	1,148,819 (72.80%)	949,779 (60.19%)	429,270 (27.20%)	1,578,089 (100%)
PlasmaUM692B_S1	2,075,713 (85.50%)	1,895,158 (78.06%)	352,030 (14.50%)	2,427,743 (100%)
PlasmaUM636SCC_S4	2,870,619 (87.69%)	2,630,572 (80.36%)	403,006 (12.31%)	3,273,625 (100%)
PlasmaUM705mAC_S3	679,258 (62.86%)	629,270 (58.24%)	401,288 (37.14%)	1,080,546 (100%)
PlasmaUM764B-IDP10_S3	477,496 (76.58%)	467,585 (74.99%)	145,993 (23.42%)	623,489 (100%)
PlasmaUM652B_S3	205,973 (47.38%)	183,607 (42.23%)	228,777 (52.62%)	434,750 (100%)
PlasmaUM686AC_S4	2,218,644 (88.75%)	2,082,164 (83.29%)	281,270 (11.25%)	2,499,914 (100%)
UM701-2AC-IDP7_S1	551,080 (67.29%)	538,818 (65.80%)	267,826 (32.71%)	818,906 (100%)
PlasmaUM735AC_S3	2,230,443 (76.27%)	1,969,870 (67.36%)	693,791 (23.73%)	2,924,234 (100%)
PlasmaUM656B_S4	397,266 (46.39%)	379,001 (44.25%)	459,155 (53.61%)	856,421 (100%)
PlasmaUM785B-IDP11_S4	454,804 (78.11%)	435,954 (74.87%)	127,455 (21.89%)	582,259 (100%)
PlasmaUM547B_S2	209,372 (19.15%)	195,717 (17.90%)	883,864 (80.85%)	1,093,236 (100%)
UM726B_S2	455,967 (53.13%)	430,805 (50.20%)	402,252 (46.87%)	858,219 (100%)
PlasmaUM662B_S4	387,393 (53.50%)	373,376 (51.56%)	336,697 (46.50%)	724,090 (100%)
UM774B_S3	627,317 (69.54%)	611,067 (67.74%)	274,762 (30.46%)	902,079 (100%)
PlasmaUM635SCC_S3	1,445,462 (76.62%)	1,333,649 (70.70%)	441,013 (23.38%)	1,886,475 (100%)
UMs709AC-IDP10_S1	338,827 (49.39%)	320,520 (46.72%)	347,252 (50.61%)	686,079 (100%)
PlasmaUM693B_S1	2,437,623 (87.69%)	2,085,236 (75.02%)	342,091 (12.31%)	2,779,714 (100%)
VA700B_S1	844,274 (61.31%)	731,733 (53.14%)	532,767 (38.69%)	1,377,041 (100%)
PlasmaUM690B_S2	1,220,428 (78.01%)	1,158,474 (74.05%)	343,932 (21.99%)	1,564,360 (100%)
PlasmaUM645B_S1	1,456,638 (81.31%)	1,283,514 (71.64%)	334,912 (18.69%)	1,791,550 (100%)
PlasmaUM748AC_S4	1,982,709 (86.18%)	1,855,694 (80.66%)	317,922 (13.82%)	2,300,631 (100%)
UM680SCC-IDP01_S1	805,107 (56.85%)	766,096 (54.09%)	611,134 (43.15%)	1,416,241 (100%)
UM751SCC-IDP09_S3	903,036 (63%)	868,586 (60.60%)	530,332 (37%)	1,433,368 (100%)
PlasmaUM727B_S2	2,391,856 (84.03%)	1,985,762 (69.76%)	454,609 (15.97%)	2,846,465 (100%)
PlasmaUM715B-IDP4_S2	1,771,627 (83.18%)	1,579,302 (74.15%)	358,316 (16.82%)	2,129,943 (100%)
PlasmaUM719B_S2	2,019,080 (83.18%)	1,678,275 (69.14%)	408,372 (16.82%)	2,427,452 (100%)
PlasmaUM777B_S4	691,396 (65.76%)	681,075 (64.78%)	359,964 (34.24%)	1,051,360 (100%)

PlasmaUM708AC_S3	2,223,966 (84.21%)	1,655,873 (62.70%)	416,970 (15.79%)	2,640,936 (100%)
UMs757B-IDP5_S2	649,742 (66.41%)	618,494 (63.22%)	328,566 (33.59%)	978,308 (100%)
PlasmaUM717AC_S1	1,107,019 (62.55%)	1,083,231 (61.20%)	662,831 (37.45%)	1,769,850 (100%)
PlasmaUM633B_S3	458,808 (52.02%)	444,119 (50.36%)	423,167 (47.98%)	881,975 (100%)
PlasmaUM786SCC_S5	396,702 (64.40%)	386,713 (62.77%)	219,330 (35.60%)	616,032 (100%)
PlasmaUM674AC_S1	478,917 (71.76%)	466,721 (69.93%)	188,447 (28.24%)	667,364 (100%)
UM759B-IDP12_S2	1,421,543 (68.97%)	1,390,874 (67.48%)	639,503 (31.03%)	2,061,046 (100%)
PlasmaUM658SCC_S3	4,713,021 (91.66%)	4,145,097 (80.61%)	428,847 (8.34%)	5,141,868 (100%)
UM741B_S4	688,693 (61.27%)	656,539 (58.41%)	435,292 (38.73%)	1,123,985 (100%)
PlasmaUM676B_S3	469,791 (69.28%)	462,424 (68.20%)	208,271 (30.72%)	678,062 (100%)
PlasmaUM642SCC_S4	1,952,169 (80.24%)	1,638,716 (67.36%)	480,624 (19.76%)	2,432,793 (100%)
UMs766AC-IDP8_S3	620,515 (65.92%)	595,086 (63.22%)	320,790 (34.08%)	941,305 (100%)
UMs761SCC-IDP7_S3	932,185 (64.49%)	822,003 (56.87%)	513,286 (35.51%)	1,445,471 (100%)
PlasmaUM667SCC_S4	2,108,957 (77.86%)	1,828,245 (67.50%)	599,618 (22.14%)	2,708,575 (100%)
PlasmaUM373B_S4	677,735 (72.80%)	664,368 (71.36%)	253,209 (27.20%)	930,944 (100%)
PlasmaUM696SCC-IDP1_S1	773,325 (76.84%)	710,985 (70.64%)	233,147 (23.16%)	1,006,472 (100%)
UM760B-IDP02_S1	2,122,653 (81.20%)	2,071,001 (79.23%)	491,320 (18.80%)	2,613,973 (100%)
PlasmaUM385B_S4	920,039 (80.67%)	891,634 (78.18%)	220,415 (19.33%)	1,140,454 (100%)
PlasmaUM641B_S1	2,739,038 (90.82%)	2,593,720 (86.01%)	276,709 (9.18%)	3,015,747 (100%)
PlasmaUM720SCC_S3	1,590,274 (82.39%)	1,290,894 (66.88%)	339,835 (17.61%)	1,930,109 (100%)
PlasmaUM730AC_S2	3,004,193 (86.79%)	2,628,896 (75.95%)	457,351 (13.21%)	3,461,544 (100%)
PlasmaUM737AC_S2	2,553,435 (80.12%)	2,410,971 (75.65%)	633,503 (19.88%)	3,186,938 (100%)
PlasmaUM734SCC-IDP7_S4	430,365 (65.97%)	409,768 (62.81%)	222,041 (34.03%)	652,406 (100%)
PlasmaUM644B_S1	3,264,500 (90.05%)	2,881,601 (79.49%)	360,573 (9.95%)	3,625,073 (100%)
PlasmaUM745SCC-IDP9_S2	506,134 (67.75%)	490,949 (65.72%)	240,915 (32.25%)	747,049 (100%)
PlasmaUM728SCC-IDP6_S3	2,077,180 (89.28%)	1,976,822 (84.97%)	249,332 (10.72%)	2,326,512 (100%)
UM740B_S3	547,615 (56.89%)	536,532 (55.73%)	415,055 (43.11%)	962,670 (100%)
PlasmaUM742B_S2	2,890,938 (89.21%)	2,488,589 (76.79%)	349,710 (10.79%)	3,240,648 (100%)
PlasmaVA525SCC_S4	223,683 (30.37%)	215,010 (29.19%)	512,834 (69.63%)	736,517 (100%)
PlasmaVA530SCC_S1	217,405 (28.54%)	210,569 (27.64%)	544,467 (71.46%)	761,872 (100%)
PlasmaUM659AC_S3	2,694,674 (91.24%)	2,522,405 (85.40%)	258,822 (8.76%)	2,953,496 (100%)
PlasmaVA473SCC_S1	827,024 (45.55%)	762,215 (41.98%)	988,615 (54.45%)	1,815,639 (100%)
PlasmaUM661AC_S4	780,368 (68.03%)	653,157 (56.94%)	366,648 (31.97%)	1,147,016 (100%)
PlasmaUM767B_S2	217,837 (50.30%)	212,486 (49.07%)	215,200 (49.70%)	433,037 (100%)

**Supplementary Table S6.** miRNAs associated with age, race, gender, and smoking status using ANOVA test		
miRNAs	Variables	p-value
miR-200a-3p	Age	<0.0001
miR-1299	Gender	0.0492
miR-215-5p	Gender	0.0368
miR-1299	Race	0.048
miR-215-5p	Race	0.0045
miR-200a-3p	Race	<0.0001
miR-215-5p	Smoking	0.0133
miR-205-5p	Stages	0.0028

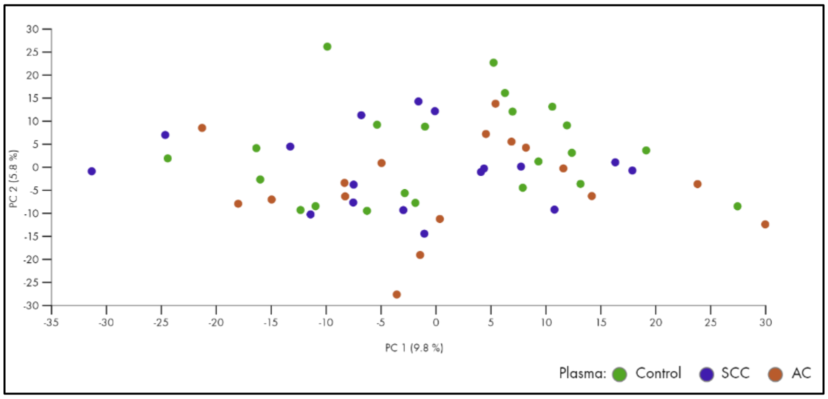

Supplemental Figure S1. Assessing inter- and intragroup variability among AC, SCC, and control samples by PCA plot. Principal components 1 and 2 for variance in miRNA expression in AC, SCC and control were displayed.

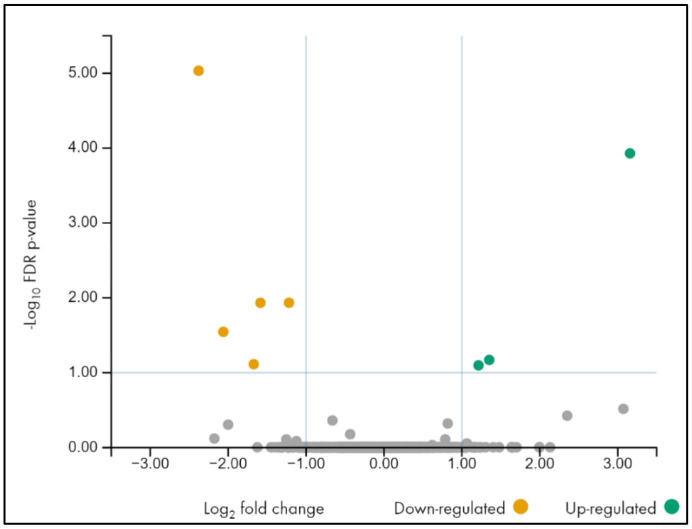

Supplemental Figure S2. Volcano plots of differentially expressed genes between NSCLC and non-cancer control. Up or down regulated miRNA were either in green or orange. The cutoff values were fold change greater than 2, p-value<0.05 and an FDR p-value ≤0.1.

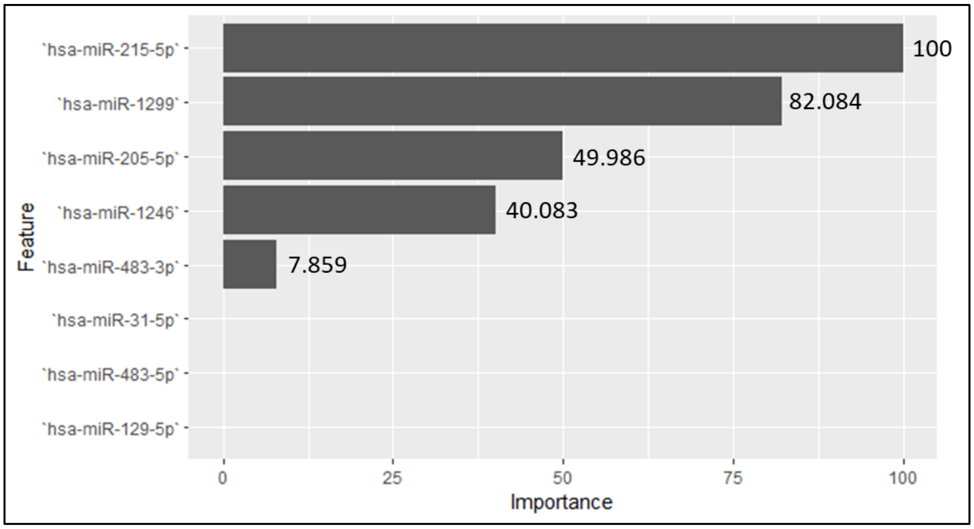

Supplemental Figure S3 The important of individual DEmiRNAs in the diagnosis of NSCLC over non-cancer control. A combination of 5 miRNAs as diagnostic biomarkers of NSCLC over non-cancer control were identified by LASSO methods.

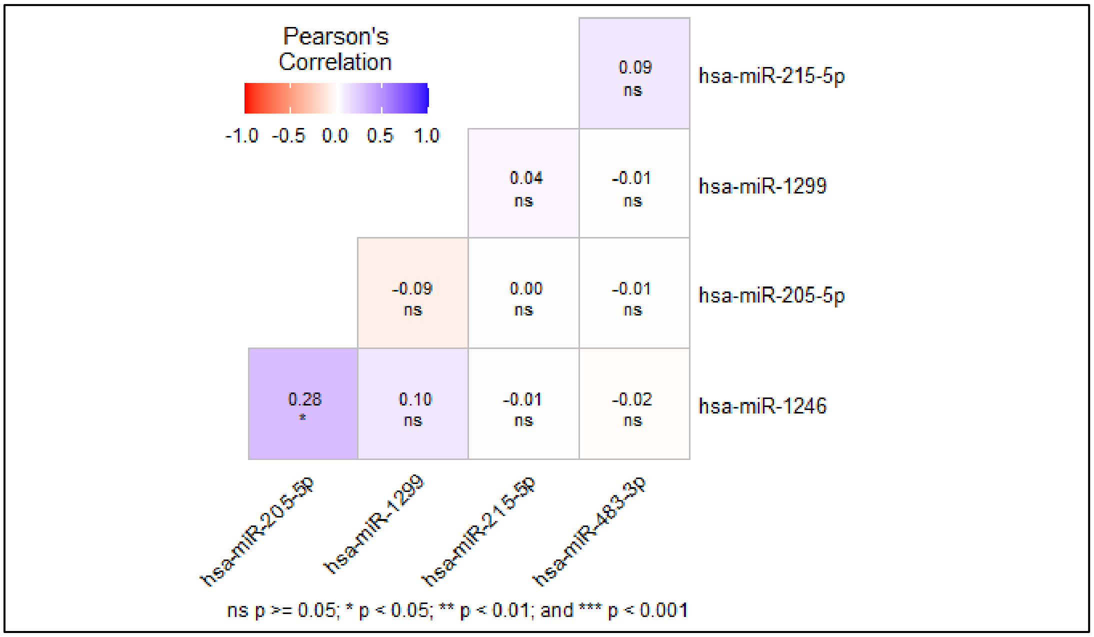

Supplemental Figure S4. No collinearity was observed among the 5 DEmiRNAs between NSCLC and non-cancer control. Pearson correlation of the 5 miRNAs were examined using R studio.

Supplemental table S7. Pearson's correlation analysis of the 5 DEmiRNAs identified by LASSO regression between NSCLC and non-cancer control.

	has-miR-1246		has-miR-1299		has-miR-205-5p		has-miR-215-5p		hsa-miR-483-3p	
	CC *	*p*	CC	*p*	CC	*p*	CC	*p*	CC	*p*
has-miR-1246	1	0.000	0.10188	0.398	0.28369	0.0165	-0.01016	0.933	-0.01793	0.882
has-miR-1299	0.1019	0.3979	1	0.000	-0.08554	0.4781	0.04065	0.736	-0.00959	0.937
has-miR-205-5p	0.2837	0.0165	-0.08554	0.478	1	0.000	0.00372	0.975	-0.00558	0.963
has-miR-215-5p	-0.0102	0.9330	0.04065	0.736	0.00372	0.9754	1	0.000	0.09353	0.438
hsa-miR-483-3p	-0.0179	0.8820	-0.00959	0.937	-0.00558	0.9631	0.09353	0.438	1	0.000

* CC: correlation coefficient. *p*-values <0.05 was considered statistically significant.

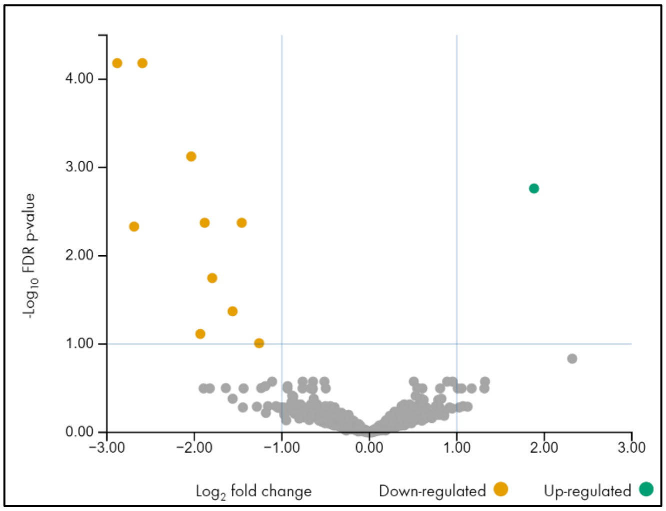

Supplemental Figure S5. Volcano plots of differentially expressed genes between SCC and non-cancer control. Up or down regulated miRNA were either in green or orange. The cutoff values were fold change greater than 2, p-value<0.05 and an FDR p-value ≤0.1.

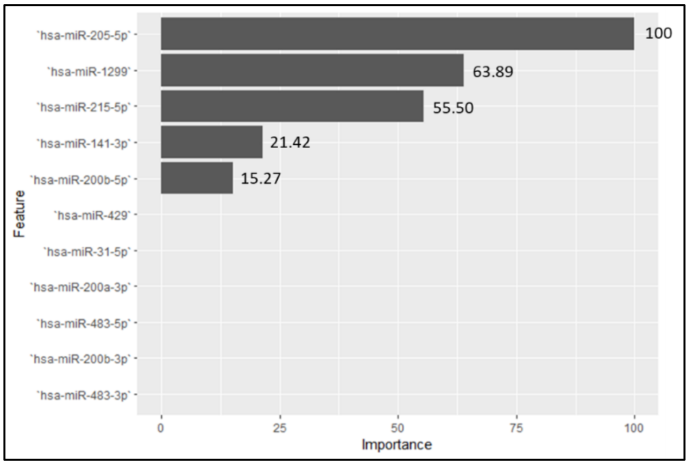

Supplemental Figure S6. Individual DEmiRNAs in the diagnosis of SCC over non-cancer control.
A combination of 5 miRNAs as diagnostic biomarkers of SCC over non-cancer control were identified by LASSO methods.

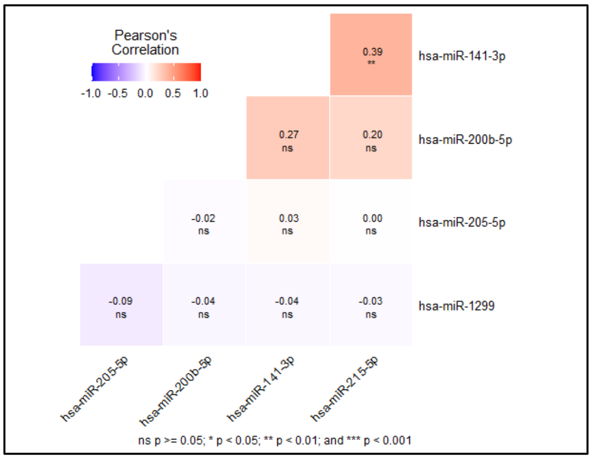

Supplemental Figure S7 No collinearity was observed among the 5 miRNAs. Pearson correlation of the 5 miRNAs were examined using R studio.

Supplemental table S8. Pearson's correlation analysis of the 5 DEmiRNAs identified between SCC and control by LASSO regression.

	has-miR-1299		has-miR-141-3p		has-miR-200b-5p		has-miR-205-5p		has-miR-215-5p	
	CC *	*p*	CC	*p*	CC	*p*	CC	*p*	CC	*p*
has_miR_1299	1	0	0.0352	0.80408	-0.0387	0.7856	-0.08785	0.536	-0.02756	0.84624
has_miR_141_3p	-0.0352	0.804	1	0	0.2660	0.0566	0.02992	0.833	0.38596	0.00471
has_miR_200b_5p	-0.0387	0.786	0.2660	0.05660	1	0	-0.01674	0.906	0.20442	0.14604
has_miR_205_5p	-0.0878	0.536	0.0299	0.83321	-0.0167	0.9062	1	0	-0.00297	0.98333
has_miR_215_5p	-0.0276	0.846	0.3860	0.00471	0.2044	0.1460	-0.00297	0.983	1	0

* CC: correlation coefficient. *p*-values <0.05 was considered statistically significant.

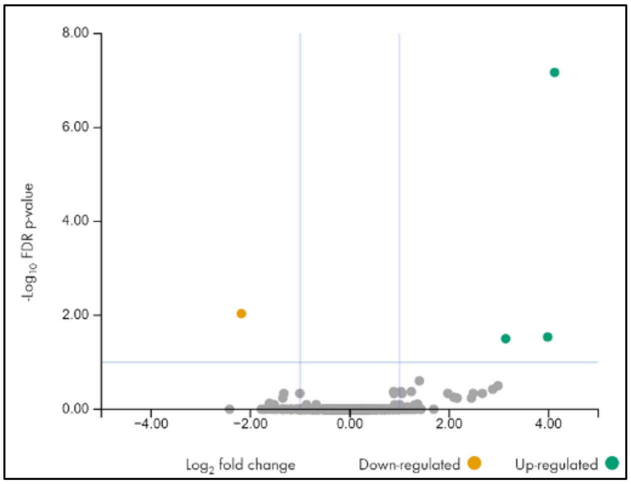

Supplemental Figure S8. Volcano plots showed four DEmiRNAs between AC and non-cancer control plasma samples identified by Geneglobe RNAseq Analysis Portal. Up- and downregulated miRNAs are shown in either green or yellow. Cutoff values were an absolute value of fold change ≥ 2, an FDR p-value ≤ 0.1 and a p-value < 0.5

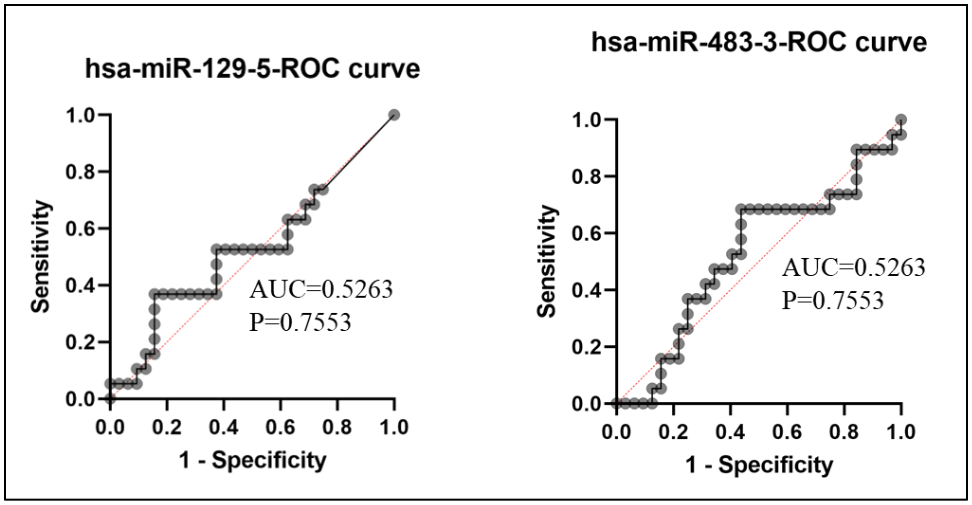

Supplemental Figure S9. Simple logistic regression and ROC curve analysis of the diagnostic power of individual DEmiRNAs in discriminating AC from non-cancer control. The correlation of sensitivity and specificity of each DEmiRNA was presented along with the Area under the curve (AUC) and p-value. Analysis and graph made by Prism Graphpad.

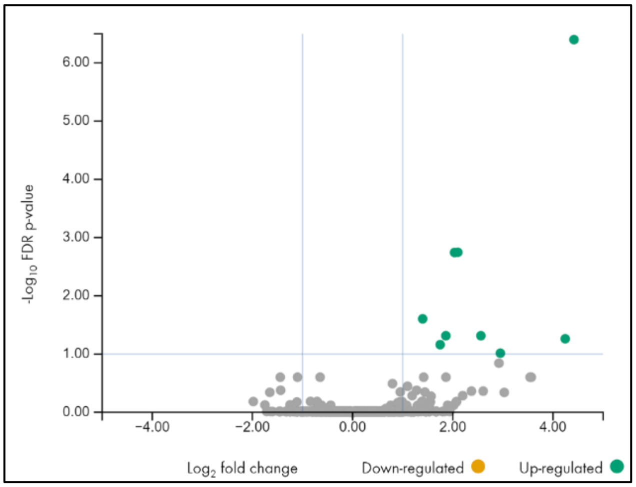

Supplemental Figure S10. A volcano plot illustrated DEmiRNAs between AC vs. SCC identified by Geneglobe
Up and downregulated miRNAs are shown in either green or yellow. Each data point represents a plasma sample. Cutoff values were an absolute value of fold change ≥ 2, an FDR p-value ≤ 0.1 and a p-value < 0.5

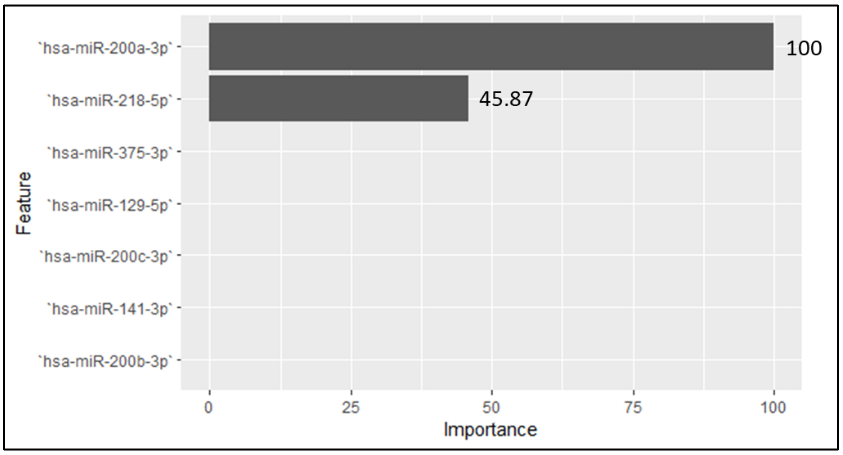

Supplemental Figure S11. The important of individual DEmiRNAs in the diagnosis of AC over SCC.
A combination of 2 miRNAs as diagnostic biomarkers of AC from SCC were identified by LASSO analysis.

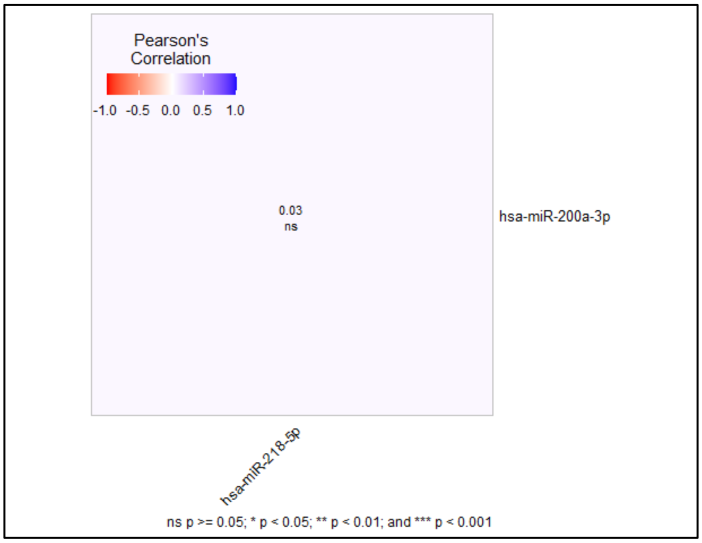

Supplemental Figure S12. No collinearity was observed among the 5 miRNAs. Pearson correlation of the 5 miRNAs were examined using R studio.

Supplemental table S9. Pearson's correlation analysis of the 2 DEmiRNAs identified between AC and SCC by LASSO regression.

	hsa-miR-200a-3p		hsa-miR-218-5p	
	CC*	*p*	CC	*p*
hsa-miR-200a-3p	1.0000	0.000	0.0336	0.839
hsa-miR-218-5p	0.0336	0.839	1.0000	0.000

* CC: correlation coefficient. *p*-values <0.05 was considered statistically significant.
